# Origin of Stability in the Solid Electrolyte Interphase formed between Lithium and Lithium Phosphorus Oxynitride

**DOI:** 10.1021/acs.chemmater.5c00483

**Published:** 2025-04-25

**Authors:** Stephen J. Turrell, Yi Liang, Tiancheng Cai, Ben Jagger, Mauro Pasta

**Affiliations:** † Department of Materials, University of Oxford, Parks Road, Oxford OX1 3PH, U.K.; ‡ The Faraday Institution, Quad One, Becquerel Avenue, Harwell Campus, Didcot OX11 0RA, U.K.

## Abstract

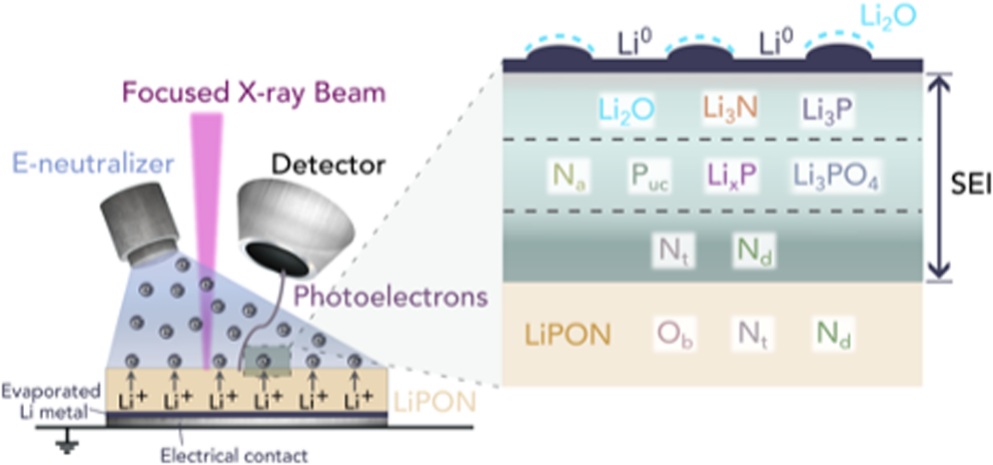

Lithium phosphorus oxynitride (LiPON) is one of the few solid electrolytes that form a truly passivating solid electrolyte interphase (SEI) when in contact with metallic lithium. Investigations into the origin of this stability may provide the insights needed to replicate it in the SEIs of alternative solid electrolyte materials. In this study, we used in situ lithium plating X-ray photoelectron spectroscopy (XPS) to investigate the formation and evolution of the Li-LiPON SEI. We show that the SEI is chemically and structurally inhomogeneous, with the fully reduced compounds identified in previous studies (Li_2_O, Li_3_N, and Li_3_P) concentrated near the lithium metal side and partially lithiated species, including Li_*x*_P, predominant closer to the LiPON side. Li_3_P and Li_*x*_P have recently been suggested as enablers of continuous SEI growth in thiophosphate solid electrolytes. We suggest that the stability of the Li-LiPON SEI is derived from a combination of the LiPON reduction potential (0.68 V vs Li^+^/Li), which is below the oxidation potentials of the fully reduced SEI compounds, and the graded structure of the SEI, which ensures that the most reduced species are not in physical or electrical contact with the LiPON layer.

## Introduction

The ongoing electrification of transport requires battery-powered vehicles with longer driving ranges and shorter charging times. However, commercial lithium-ion batteries, which rely on organic liquid electrolytes and graphite-based anodes, cannot provide the levels of specific energy and charging rate capability needed to achieve these performance improvements. While metallic lithium has a specific capacity over 10 times higher than that of graphite, standard organic liquid electrolytes are unstable and raise safety concerns when paired with a lithium metal anode, meaning that alternative electrolytes are required.

A revival of interest in all-solid-state batteries has occurred in recent years, driven by the expectation that certain inorganic solid electrolyte materials will solve the issues of poor electrochemical stability and short-circuiting by lithium dendrites that derailed early attempts to develop rechargeable lithium metal batteries.^1−3^ Although the Li^+^ conductivities of early solid electrolytes were too low for most practical applications, values comparable to those of organic liquid electrolytes (∼10^–2^ S cm^–1^) have since been achieved.^4−7^ Unfortunately, the electrochemical stability in contact with lithium metal and resistance to dendrite penetration of these materials are poorer than originally anticipated, precluding the fabrication of all-solid-state cells with satisfactory performance for most applications.^8,9^ The formation of lithium dendrites on charging is largely attributable to microstructural flaws created within the solid electrolyte during processing, which propagate as they fill with lithium, whereas the poor electrochemical stability at the interface with lithium metal is an intrinsic property of most solid electrolyte materials investigated to date.

Although a solid electrolyte with both a high Li^+^ conductivity and adequate stability in contact with lithium metal has yet to be found, a material with a relatively low Li^+^ conductivity but outstanding stability in contact with lithium metal—lithium phosphorus oxynitride—has been studied for over 30 years. This glassy material was originally reported by Marchand in 1983 and identified as a viable solid electrolyte by Bates et al. in 1992.^10,11^ Bates et al. fabricated this material, which they referred to as “LiPON”, by the reactive radio frequency (RF) magnetron sputter deposition of Li_3_PO_4_ in a nitrogen atmosphere, producing films with thicknesses on the order of 1 μm. While the ∼10^–6^ S cm^–1^ Li^+^ conductivity of LiPON is low compared to values reported for many powder-processed solid electrolytes, the relative thinness of a typical LiPON film ensures that its impedance is not significantly higher than that of most “bulk” electrolytes. Crucially, LiPON is electrochemically stable in contact with lithium metal and has demonstrated exceptional resistance to lithium dendrite penetration in thin film cells.^12−14^ In a noteworthy study, Neudecker et al. showed that a “lithium-free” cell with a 2 μm thick LiPON electrolyte and 2.7 μm thick LiCoO_2_ cathode remained stable for over 500 cycles between 3.0 and 4.2 V vs Li^+^/Li at 5 mA cm^–2^.^15^ While LiPON is only viable as a solid electrolyte for low-capacity thin film cells, understanding the reasons for its unparalleled electrochemical and mechanical stability will aid the development of bulk solid electrolytes with satisfactory all-round performance.

The focus of our investigation was the electrochemical stability of the Li|LiPON interface. In a series of studies between 2013 and 2016, the Janek research group developed a new in situ lithium deposition X-ray photoelectron spectroscopy (XPS) technique to investigate the stabilities of interfaces between solid electrolytes and lithium metal.^16−19^ The technique involves the sputter deposition of lithium metal layers onto the solid electrolyte from an adjacent lithium foil using an argon ion gun, which is a feature of most XPS systems. Sputter deposition and acquisition of XPS spectra are performed alternately to track the chemical changes at the sample surface with minimal perturbation from the atmosphere. After applying the technique to a variety of Li^+^-conducting solid electrolytes including Li_1+*x*_Al_*x*_Ge_2–*x*_(PO_4_)_3_ (LAGP), Li_0.35_La_0.55_TiO_3_ (LLTO) and Li_10_GeP_2_S_12_ (LGPS), the group suggested that the interfaces formed should be categorized into three types depending on their stability: (1) thermodynamically stable, (2) thermodynamically unstable with a mixed conducting interphase (MCI), and (3) thermodynamically unstable with a kinetically stabilized interphase. For types (2) and (3), the thermodynamic instability of the solid electrolyte in contact with lithium metal results in the formation of a layer of reaction products at the interface—an interphase.

The extent to which an interphase grows depends on its ability to conduct Li^+^ and electrons. A high Li^+^ conductivity is desirable to minimize the internal impedance of the cell, but if the electronic conductivity is also high (as in an MCI), Li^+^ and electrons from the anode continue to reach the solid electrolyte as the interphase thickens, allowing a continuous reaction until the electrolyte has been consumed. If the electronic conductivity is low, the supply of electrons to the solid electrolyte declines significantly as the interphase thickens, inhibiting the decomposition reaction and eventually passivating the interface. In this case, the interphase is known as a solid electrolyte interphase (SEI) after the equivalent layer in liquid electrolyte cells that has been studied for several decades.^20^ A thermodynamically stable interface is rare because lithium metal tends to reduce most cations and react with oxygen and sulfur to form Li_2_O and Li_2_S. Therefore, when interfacial stability is observed, it is usually the result of limited degradation reaction kinetics rather than thermodynamic stability.^16^

Indeed, in 2015, Schwöbel et al. used XPS to show that an SEI consisting of Li_2_O, Li_3_N, and Li_3_P formed when lithium metal was evaporated onto a LiPON film.^21^ Sicolo et al. reanalyzed the XPS spectra in a follow-up study and found evidence that Li_3_PO_4_ was also present in the SEI.^22^ The thermodynamic instability of the Li|LiPON interface was confirmed by Zhu et al., who used first-principles calculations to show that the theoretical stability window of LiPON is 0.68–2.63 V vs Li^+^/Li.^23^ Together with the experimental evidence, this suggests that the apparent electrochemical stability of LiPON in lithium metal cells is due to the formation of passivating interphases with the electrodes.

More recently, two transmission electron microscopy (TEM) studies have found evidence that a 60–80 nm thick SEI containing Li_2_O and Li_3_N forms at the Li|LiPON interface.^24,25^ However, they disagreed over the nature of the P-containing compound: the cryogenic high-resolution TEM study on a lift-out from a Li|LiPON interface by Cheng et al. only found evidence for the presence of Li_3_PO_4_, while the P^3–^ peaks in electron energy loss spectra collected by Hood et al. from a Li|LiPON interface formed in situ indicated the presence of Li_3_P. Nevertheless, both studies came to similar conclusions regarding the spatial distribution of the SEI components: the P-rich phase (Li_3_PO_4_ or Li_3_P) was concentrated at the LiPON side of the interphase, while the O-rich phase (Li_2_O) was predominant at the lithium metal side. The recent first-principles calculations of interfacial stability between these SEI components and lithium metal by Wang et al. provided an explanation for this: Li|Li_2_O was found to be the most stable interface and Li|Li_3_PO_4_ the least stable followed by Li|Li_3_P,^26^ while Li|Li_3_N had an intermediate stability.

The Li-LiPON SEI has also been characterized in electroanalytical and neutron reflectometry studies on (metal current collector)|LiPON|Li cells at Oak Ridge National Laboratory by Westover et al. and Browning et al., respectively.^27,28^ By equating the irreversible capacity loss during the plating of lithium on the current collector (accounting for contributions from known secondary loss mechanisms) with the lithium consumed by reaction with LiPON to form the SEI, the Li-LiPON interphase was found to be on the order of 5 nm thick—significantly thinner than reported by the TEM-based studies. Furthermore, neutron reflectometry measurements confirmed that the SEI thickness was less than 7 nm, highlighting the significant disparity between the measurements of SEI thickness made using TEM and other techniques.

The aim of our investigation was to study the chemical and structural changes associated with SEI formation on LiPON as lithium is plated under conditions that simulate the first charge of an “anode-free” cell.^29^ These conditions were created by employing a relatively new in situ XPS technique first reported by Wood et al., which uses the electron beam generated by the built-in electron flood gun of the XPS system to plate lithium electrochemically on the LiPON surface of Li|LiPON bilayer samples.^30^ While this technique has been used to study interphase formation on Li_2_S–P_2_S_5_, Li_7_La_3_Zr_2_O_12_ (LLZO), Li_10_GeP_2_S_12_ (LGPS), and Li_6_PS_5_Cl solid electrolytes, we are unaware of any previous reports on its application to LiPON.^31−34^ The effects of LiPON processing route, composition, and thickness on SEI formation were investigated to assess the variability that can be expected between different studies and practical implementations of LiPON electrolytes. An improved understanding of the nature and formation process of the Li-LiPON SEI will aid the development of solid electrolytes with better all-round performance.

## Methods

### Preparation of Substrates for LiPON Films

Disks of 304 stainless steel (15.8 mm diameter coin cell spacers) and ∼1 cm^2^ pieces of undoped silicon cut from ⟨100⟩ oriented wafers (PI-KEM Ltd.) were used as substrates for thin film deposition. The stainless steel disks were ground and polished to achieve a mirror-like surface finish free from visible scratches; the final polish was performed with a 1 μm grade diamond suspension. All substrates were cleaned by sonication in isopropanol and distilled water, dried, and mounted on glass slides with double-sided Kapton tape prior to film deposition.

### Deposition of Thin Films

A physical vapor deposition (PVD) system (MB EVAP, MBraun) integrated into an argon-filled glovebox was used for the deposition of thin films of lithium, LiPON, and nickel. Lithium films approximately 1–2 μm thick (measured using the quartz crystal monitor of the PVD system) were deposited by the thermal evaporation of lithium granules (Glentham Life Sciences Ltd., purity 99.4%) from a molybdenum boat under vacuum. LiPON films were deposited by RF magnetron sputtering; a circular magnetron source and a target-to-substrate distance of ∼12 cm were used. Sputtering targets were prepared within the glovebox by lightly pressing dried lithium orthophosphate (Li_3_PO_4_) powder (Merck) into a 2″ diameter, ∼2 mm deep circular copper holder using a glass microscope slide. The base vacuum pressure for the deposition processes was below 5 × 10^–5^ mbar. Nitrogen gas was injected at a rate of 20 sccm for sputter deposition of the LiPON, which, along with baffling of the vacuum pumping, achieved a process pressure of ∼1 × 10^–2^ mbar. The applied RF power was 50 W, and two different thicknesses of LiPON were achieved by depositing for either 6 h or 40 min. Nickel films were required to form electrical contacts on certain LiPON samples; these were deposited by RF magnetron sputtering using an argon process gas to thicknesses of at least 200 nm. All film samples were stored in a glovebox after fabrication.

### Bulk Synthesis of LiPON

Although LiPON is rarely produced by techniques other than RF magnetron sputtering, we also synthesized it in bulk form using a method based on the small number of prior reports on this subject.^10,35−37^ 1.5 g of lithium metaphosphate (LiPO_3_) powder (Stanford Advanced Materials, purity 99.9% metals basis) was placed into an alumina boat that had been coated with boron nitride from an aerosol. The boat was positioned centrally within a quartz glass tube (22 mm inner diameter, 1.2 m length) and enclosed in a split tube furnace (Carbolite Ltd.). The tube was heated to 750 °C at a rate of 10 °C min^–1^.

During heating, nitrogen gas was flowed at a rate of ∼300 sccm. The function of this heating step was to remove moisture and melt the powder in an inert atmosphere (the melting temperature of LiPO_3_ is 656 °C).^38^ To the best of our knowledge, in all previous reports on the bulk synthesis of LiPON, the molten LiPO_3_ was cooled to form a glass and later remelted under ammonia flow to form LiPON by ammonolysis. We found that this intermediate cooling step was unnecessary and dispensed with it. Shortly before the end of the heating ramp, the nitrogen supply was turned off; the nitrogen flow continued for several minutes as the pressure at the flow meter inlet equalized with that at the tube furnace outlet. Once the nitrogen flow had ceased, a flow of anhydrous ammonia (BOC Ltd.) was initiated at a rate of ∼200 sccm; the transition of the tube atmosphere from nitrogen to ammonia was gradual as it took several minutes for the residual nitrogen to be displaced.

The temperature of the furnace was held at 750 °C for 3 h and 15 min, and the flow of ammonia was maintained until the final 15 min of this dwell period. During the first 3 h of the dwell period, ammonolysis of the LiPO_3_ occurred by eq 1.
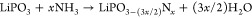
1

After the flow of ammonia was turned off, the gas remaining in the system was allowed to flow through the furnace tube before the nitrogen flow was restarted. We found that the brief annealing period (5–10 min) under a pure nitrogen atmosphere at 750 °C—which was not implemented in the previous studies referenced above—significantly reduced the total volume of water vapor bubbles trapped within the glass after cooling. We also found that longer annealing periods did not lead to significant further reductions in bubble volume but resulted in nitrogen loss.

At the end of the 750 °C dwell, the nitrogen flow was maintained, and the furnace was allowed to cool for several hours at its natural rate until the temperature was low enough (below ∼200 °C) to remove the boat from the tube. Prior to characterization, the glass casting was broken into pieces to create a series of samples. Each sample was alternately sonicated in isopropanol and ground on successively finer silicon carbide papers to remove the boron nitride residue and create parallel-sided samples with thicknesses below 1 mm. Final polishing was performed inside an argon-filled glovebox ([H_2_O] and [O_2_] < 1 ppm) using a 1 μm grade diamond lapping film to achieve a reflective finish free of visible scratches. The samples were stored inside a glovebox until required.

### Structural Characterization

Scanning electron microscopy (SEM) was performed on thin film and bulk-processed samples of LiPON to study their surface and cross-sectional microstructures and morphologies. A Zeiss Merlin instrument operating at a 5 kV accelerating voltage, 500 pA beam current, and 8.5 mm working distance was used for this purpose. The identification of microstructural features was aided by energy-dispersive X-ray spectroscopy (EDX) using an Oxford Instruments Ultim Max 170 X-ray detector. LiPON films deposited on undoped silicon were selected for SEM characterization, as they could be sectioned with the aid of a diamond scribe to create pristine fracture surfaces for cross-sectional imaging. Samples were transferred from the glovebox to the SEM instrument using an airtight transfer holder (Gatan, Inc.).

The thickness of LiPON deposited for 6 h was measured from a cross-sectional secondary electron micrograph, while that of each bulk-processed sample was measured using calipers. However, the LiPON deposited for 40 min was too thin to resolve clearly by SEM, so atomic force microscopy (AFM) was used to measure its thickness. A piece of Kapton tape was used to cover part of an undoped silicon substrate prior to LiPON deposition; when this tape was removed after deposition, a clean film edge was created. The ScanAsyst-Air probe of a Bruker Dimension Icon instrument was scanned across this edge in ScanAsyst mode at a rate of 0.5 Hz. Measurements were performed within an Ar-filled glovebox ([H_2_O] and [O_2_] < 1 ppm).

### Ionic Conductivity Measurements

Electrochemical impedance spectroscopy (EIS) was performed on a LiPON film deposited for 6 h on a stainless steel substrate and a sample of bulk-processed LiPON. Prior to these measurements, pieces of Kapton tape containing a punched 2 mm diameter hole were attached to the top surface of the thin film sample and the parallel surfaces of the bulk-processed sample (aligned to coincide). Nickel electrical contacts were then sputter-deposited; the Kapton tape provided the required shadow masking and was subsequently removed. To prevent air exposure during the EIS measurements, each sample was inserted into a laminated aluminum pouch, connected to copper foil electrodes using Kapton tape, and sealed under vacuum within an Ar-filled glovebox ([H_2_O] and [O_2_] < 1 ppm). Standard materials and methods for laboratory-scale pouch cell construction were used.

The pouches were removed from the glovebox and connected to an MTZ-35 impedance analyzer (BioLogic Science Instruments). Impedance measurements were performed from 35 MHz to 0.1 Hz at an amplitude of 10 mV. On account of its novelty, additional impedance measurements were performed on the bulk-processed LiPON sample at a series of temperatures between 25 and 100 °C for the determination of the activation energy. A temperature-controlled climatic chamber (ITS, BioLogic Science Instruments) was used to bring the sample to the required temperatures. Equivalent circuit models were fitted to the impedance data using ZView software (Scribner Associates, Inc.) in order to determine the ionic resistance of each LiPON sample, thereby enabling calculations of ionic conductivity.

### Chemical Characterization and Study of SEI Formation

XPS characterization of thin film and bulk-processed LiPON samples was performed in a Physical Electronics (PHI) VersaProbe III instrument with an Al Kα (*h*ν = 1486.6 eV) source and a chamber maintained at a pressure below 1 × 10^–8^ mbar. Each sample to be characterized was attached in turn to the XPS sample holder within an Ar-filled glovebox ([H_2_O] and [O_2_] < 1 ppm) using electrically conductive carbon tape and transferred to the XPS instrument in an airtight vessel (PHI Transfer Vessel). X-ray-induced secondary electron imaging (SXI) was used to locate the center of the sample, and the probing area for the XPS measurements was set to 500 μm × 500 μm about this point.

**Figure 1:**
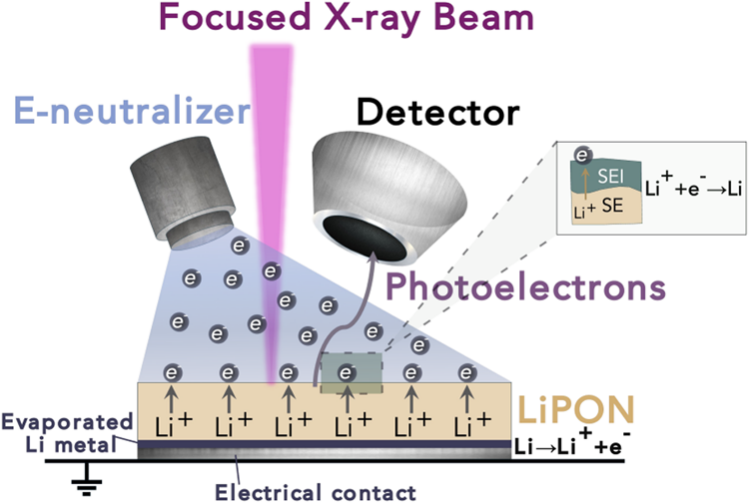
Schematic diagram outlining the operating principle of the in situ lithium plating XPS technique used in this investigation. A layer of lithium underneath the LiPON acted as the lithium source; this was electrochemically driven to the LiPON surface by an electron beam generated by the electron flood gun of the XPS instrument. Lithium deposition was performed in a stepwise fashion and was alternated with the acquisition of XPS spectra to characterize the chemical and structural changes at different stages of SEI formation.

Prior to performing XPS measurements on the pristine samples, the surfaces were etched for 1 min using the built-in Ar-ion gun operating at 2 kV to remove most of the surface contamination without materially altering the LiPON structure. The pass energy for the high-resolution XPS scans was 55 eV, and the duration of each scan was approximately 33 min. CasaXPS software (Casa Software Ltd.) was used for data processing: a Shirley background was applied to each core-level spectrum, peak components were fitted using a Gaussian–Lorentzian line shape, and binding energy values were calibrated using the adventitious C 1s spectral component at 284.8 eV.^39^ Chemical compositions were determined using relative sensitivity factors calculated for our instrument at the same pass energy (Table S1). The chemical compositions (excluding lithium) were verified using EDX. After the LiPON surfaces were characterized, in situ lithium plating XPS was performed to study the development of the SEI between LiPON and lithium metal. A schematic diagram outlining the operating principle of this technique is shown in Figure 1.

The in situ lithium plating technique requires a source of lithium beneath the LiPON. For the thin film samples, this was achieved by evaporating an ∼1 μm layer of lithium onto stainless steel substrates prior to sputter depositing the LiPON. For the sample of bulk-processed LiPON, an ∼2 μm layer of lithium was evaporated onto one of the parallel surfaces followed by the sputter deposition of ∼350 nm of nickel. The function of the nickel layer was to protect the underlying lithium and improve its electrical contact with the XPS sample holder. To perform the in situ plating, the electron flood gun of the XPS instrument was directed at the sample and used to apply a current of 30 μA. The diameter of the electron beam at the LiPON surface was approximately 5 mm, but the definition of this current path was improved by covering each sample with Kapton tape containing a punched 5 mm diameter hole. Therefore, the nominal applied current density was ∼0.15 mA cm^–2^, which is typical of values used previously for cycling all-thin-film cells with LiPON electrolytes.^12−15^

**Figure 2:**
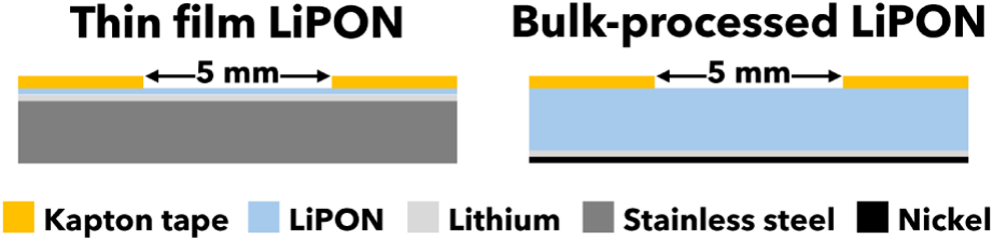
Schematic diagrams showing cross sections of the samples fabricated for in situ lithium plating XPS experiments. A thermally evaporated film of lithium beneath the LiPON acted as the source for lithium plating. Punched Kapton tape was placed on top of the samples to define a 5 mm diameter circular current path.

As electrons accumulated on the LiPON, the resulting potential difference initiated the oxidation of the underlying lithium to Li^+^. This was drawn to the surface and reduced back to lithium metal, as shown schematically in Figure 1. Cross sections of the samples used for the in situ lithium plating XPS experiments are illustrated schematically in Figure 2. The lithium metal deposited on the LiPON surface reacted with the LiPON to form an SEI. By alternating between lithium plating and XPS acquisition, it was possible to track chemical changes in the near-surface region. The electron flood gun was kept off during the acquisition of XPS spectra.

## Results and Discussion

### Preliminary Characterization of the LiPON

Prior to performing the in situ XPS experiments, samples of thin film and bulk-processed LiPON were characterized to confirm their morphological, microstructural, and electrical properties. Photographs of the bulk-processed LiPON immediately after synthesis and after grinding and polishing are shown in Figure S1a and b, respectively. Figure S2a and b show secondary electron micrographs of a LiPON film sputter-deposited on undoped silicon for 6 h, while Figure S2c and d show a ground and polished bulk-processed LiPON sample. The surface of the thin film sample in Figure S2a consisted of a smooth, featureless LiPON layer beneath a nonuniform distribution of rounded particles approximately 1 μm in diameter. EDX measurements showed that these particles were enriched in carbon and oxygen and were thus likely to have been precipitates of lithium carbonate formed during storage. A LiPON film thickness of ∼0.6 μm was measured from the cross-sectional view in Figure S2b. Figure S2c and d show that grinding the bulk-processed LiPON on silicon carbide paper was effective at removing the large bubbles created during ammonolysis. No bubbles were present in the surface region in Figure S2c, and the cross-sectional view in Figure S2d shows that the few bubbles remaining in the glass were relatively small (below 30 μm in diameter) and well dispersed.

The results of EIS measurements on an ∼0.6 μm thick LiPON film and an ∼400 μm thick bulk-processed LiPON sample are shown in Figures S3 and S4, respectively. All of the Nyquist plots display a steep tail at low frequencies, which shows that the blocking electrodes were effective and that the LiPON samples had low electronic conductivities. The Nyquist plot for the thin film sample in Figure S3a was simulated using an equivalent circuit (inset) with contributions from bulk and interfacial impedances; these contributions are shown separately in Figure S3b. The interfacial resistance, *R*, could have been due to lithium carbonate, so the bulk resistance, *R*_i_ was taken to be the ionic resistance of the LiPON film. The corresponding value of ionic conductivity was approximately 5 × 10^–7^ S cm^–1^, assuming that the LiPON thickness was the same as that measured for the film deposited on silicon. Although this is lower than the values achieved by Bates et al. (2–3.3 × 10^–6^ S cm^–1^), several studies have reported values on the order of 10^–7^ S cm^–1^, so the value reported here is within the expected range for LiPON films.^11,40,44^

A contribution from an interfacial layer is not evident in the Nyquist plots for the bulk-processed LiPON sample, as shown in Figure S4a, which could be simulated with a single semicircle. However, an interfacial contribution on the order of that seen for the thin film sample would not be resolvable since the impedance of the bulk-processed sample at 25 °C was approximately 4 orders of magnitude higher than that of the thin film sample. The ionic conductivity at 25 °C and activation energy of the bulk-processed LiPON (calculated from the Arrhenius plot in Figure S4b) were ∼2 × 10^–8^ S cm^–1^ and 0.71 eV, respectively, which are approximately within the ranges reported previously (∼6 × 10^–9^ to 3 × 10^–7^ S cm^–1^ and 0.6–0.7 eV).^35,36^ Aside from the typically lower Li^+^ concentration of bulk-processed LiPON,^35,44^ one reason for the lower ionic conductivity of the bulk-processed sample compared to the thin film sample would have been the presence of bubbles in its microstructure, which increased the tortuosity of the current path during EIS measurements.

### In Situ Lithium Plating XPS Experiments

**Figure 3:**
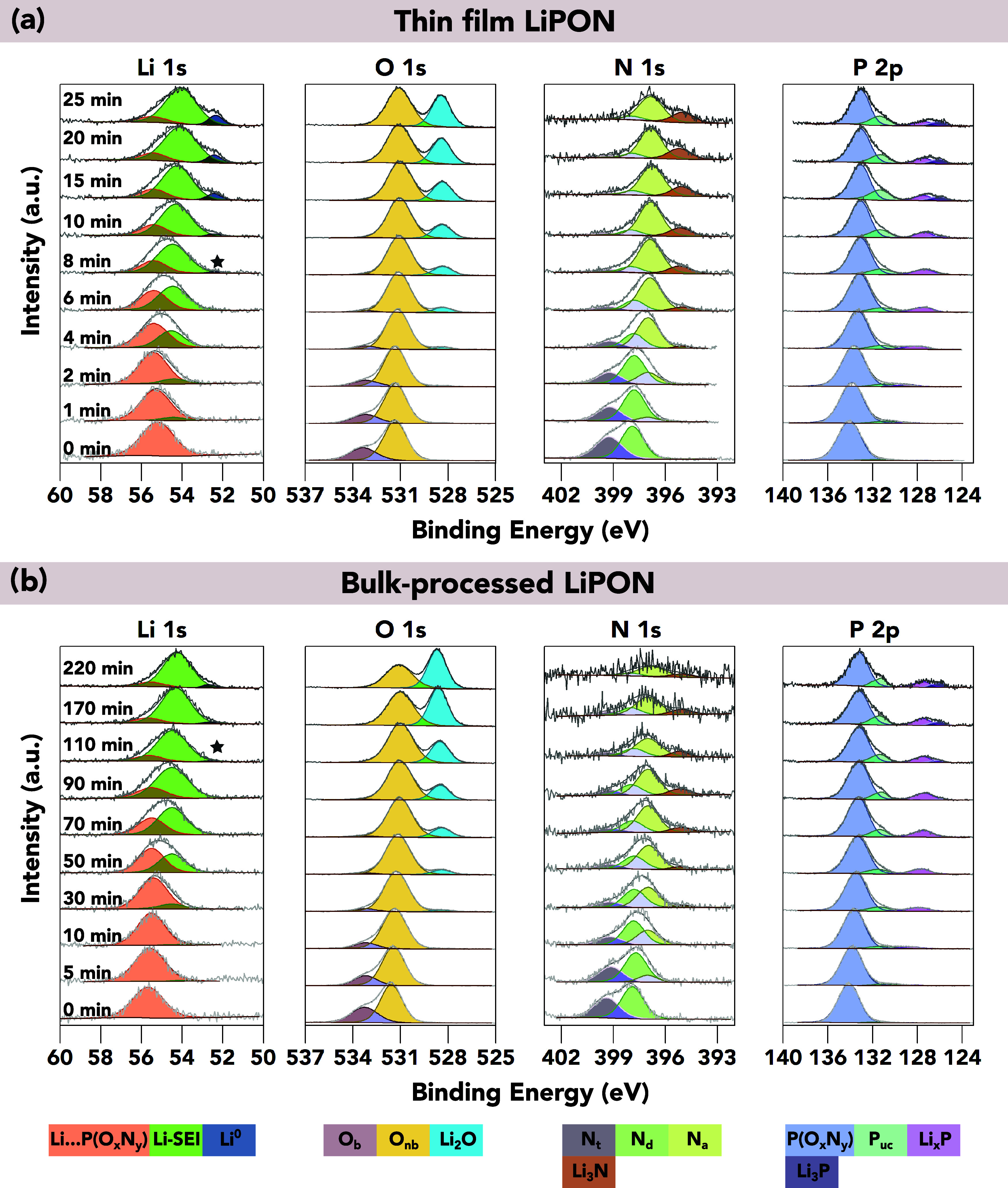
Time series of XPS core-level spectra acquired during the in situ lithium plating experiments performed on (a) an ∼0.6 μm thick LiPON film sample and (b) an ∼800 μm bulk-processed LiPON sample. Acquired spectra (gray) are shown along with linear combination fitting results; the intensities were normalized to the strongest peak in each spectrum to improve the visibility of minor spectral contributions. The cumulative lithium plating time (electron beam exposure time) is indicated in the first column. For the sake of clarity, spectra acquired at intermediate plating times are not displayed. Individual spectral components are colored, and a key is provided below each group of spectra. O_b_, O_nb_, N_t_, N_d_, N_a_, and P_uc_ are bridging oxygen, non-bridging oxygen, triple-bridging nitrogen, double-bridging nitrogen, apical (non-bridging) nitrogen, and undercoordinated phosphorus, respectively. The appearance of the Li^0^ (lithium metal) peak is highlighted with a star.

Once we had ascertained that the electrical properties of the thin film and bulk-processed LiPON samples were consistent with previous reports, we proceeded to study the structural and chemical properties at the molecular level and how they changed as lithium metal was deposited. Figure 3a and b show the XPS core-level spectra collected during in situ lithium plating experiments on an ∼0.6 μm thick LiPON film and an ∼800 μm thick sample of bulk-processed LiPON, respectively. The intensities of each acquired core-level spectrum were normalized to that of the strongest peak to improve the visibility of minor spectral contributions; the unnormalized versions of these plots are shown in Figure S5.

#### Molecular Properties Prior to Lithium Plating

The series of core-level spectra labeled “0 min” were acquired immediately after performing Ar-ion etching on the pristine samples. Figure S6 shows that the intensity of the C 1s emission from both samples was somewhat lower after etching, which suggests that the etching effectively removed a substantial amount of carbonate contamination. The etching had a negligible effect on the LiPON molecular structure, as shown by the O 1s and Li 1s spectra collected before and after etching in Figure S7. Minor changes were however seen in the N 1s and P 2p spectra, which are addressed in the following discussion.

Remarkably, the appearances of the corresponding spectra in Figure 3a and b are very similar, indicating that the molecular structures of the thin film and bulk-processed LiPON samples had similar characteristics. To some extent, this could be explained by the fact that the chemical compositions of the samples—as determined from these spectra—were rather similar in terms of Li, P, and O content, as shown in Table 1. While the N/P ratio of the thin film sample was almost double that of the bulk-processed sample, the difference narrowed slightly when an emission at 403.3 eV in the N 1s spectrum of the bulk-processed sample was included in the calculation (see Supporting Note 1). Furthermore, the N/P ratios determined by EDX (which is less surface sensitive than XPS) were somewhat higher (Table S2), particularly for the bulk-processed sample, which could either indicate an N deficiency at the film surface or reflect the fact that some of the surface N was not present in the chemical environments expected for LiPON (Supporting Note 1). The O/P ratios measured using EDX were in much better agreement with the XPS results.

**Table 1:** Chemical Compositions and Ratios of Bridging to Non-Bridging Oxygen (O_b_/O_nb_) and Triple-Bridging to Double-Bridging Nitrogen (N_t_/N_d_) Calculated for the Thin Film and Bulk-Processed LiPON Samples from the XPS Spectra Acquired Prior to Lithium Plating in Figure 3^a^

	Thin Film LiPON	Bulk-Processed LiPON
Chemical Composition	Li_1.60_PO_2.72_N_0.46_	Li_1.30_PO_2.66_N_0.25_
O_b_/O_nb_	0.37	0.49
N_t_/N_d_	0.80	0.72

a The bulk-processed LiPON composition neglects an emission in the N 1s spectrum from NO_2_^–^, which is not part of the LiPON structure. The composition calculated with NO_2_^–^ included is provided in Supporting Note 1.

In previous XPS studies on thin film and bulk-processed LiPON, the principal emissions in the O 1s and N 1s spectra were fitted with two components since they each contained an asymmetric shoulder peak.^21,22,35,45−47^ Similar fittings were performed in this study, as shown in Figure 3. Thus, O and N are each present in two distinct chemical environments. However, an additional peak in the N 1s spectrum centered at ∼404 eV was seen in previous studies and attributed to O–N=O (NO_2_^–^). As mentioned above, a minor peak at 403.3 eV was present in the N 1s spectrum from the bulk-processed sample, but not in that from the thin film. Furthermore, this peak was absent before the initial Ar-ion etching, which is shown by the extended-range N 1s spectra in Figure S8. Previous reports suggest that NO_2_^–^ is a surface phase rather than part of the LiPON structure, so it has been excluded from Figure 3 and subsequent analysis (see Supporting Note 1 for further remarks).^48,49^ Evidence for this is provided by the unnormalized N 1s spectra acquired from the thin film and bulk-processed LiPON samples during the in situ lithium plating experiments, which are shown over an extended binding energy range in Figure S9. While an NO_2_^–^ peak is absent from the spectra acquired from the thin film sample, the NO_2_^–^ peak seen after etching the bulk-processed sample decreases in intensity during lithium plating, consistent with NO_2_^–^ being a surface phase. As in the previous studies, the Li 1s and P 2p emissions were fitted with single components (the P 2p peak in fact consists of two spin–orbit components separated by 0.86 eV, which are not highlighted in Figure 3 for clarity).

Spectral components were matched to chemical environments in LiPON based on the attributions made in previous reports; examples of these environments are indicated on the structural formula of a hypothetical LiPON fragment in Figure S10. The single component at 55.2–55.7 eV in the Li 1s spectra was fitted with a component labeled “Li···P(O_*x*_N_*y*_)” since all the Li^+^ environments in LiPON are chemically similar: each Li^+^ is attached to a non-bridging oxygen or nitrogen atom of a P(O_*x*_N_*y*_) group. In the O 1s spectra, the component at higher binding energy (∼533 eV) is attributed to oxygen in P–O–P bonds, which is usually termed bridging oxygen (O_b_) since it is common to two P(O_*x*_N_*y*_) groups. The more intense component at lower binding energy (531–532 eV) is attributed to non-bridging oxygen (O_nb_). In the N 1s spectra, the component at higher binding energy (∼399 eV) is attributed to “triple-bridging N” or N_t_ (N that is common to three P(O_*x*_N_*y*_) groups), while the component at lower binding energy (∼398 eV) is attributed to “double-bridging N” or N_d_ (N that is common to two P(O_*x*_N_*y*_) groups).^50,51^ The most primitive molecules containing N_t_, N_d_, N_a_, and O_b_ are illustrated by the structural formulas in Figure S13 to aid the comparison of these bonding arrangements.

It is important to note that Lacivita et al. have called into question the assignment of the main N 1s spectral components to N_d_ and N_t_ in the case of LiPON thin films.^50^ From ab initio molecular dynamics simulations, they found that LiPON with a similar Li/P ratio to the Li_3_PO_4_ sputtering target contained nitrogen in the form of N_d_ and N_a_ (apical, non-bridging N) but not N_t_. Sicolo et al. had previously found that a simulated melt quench on crystalline Li_1.25_PO_2_N_0.75_ formed an amorphous structure containing both N_d_ and N_t_.^52^ Their LiPON composition was rather deficient in Li and O and thus closer to the LiPO_3_ precursor of bulk-processed LiPON. The results of these computational studies suggest that the formation of N_a_ over N_t_ is favored at high Li/P ratios, consistent with the fact that P(O_*x*_N_*y*_) units containing N_a_ accommodate more Li^+^ than those containing N_t_ (Figure S13). Indeed, Lacivita et al. suggested that the transition from predominantly N_t_ and N_d_ nitrogen environments to predominantly N_d_ and N_a_ environments occurs above an Li/P ratio of ∼2.2. Based on this, they concluded that the N 1s components at ∼398 and ∼399 eV in the XPS spectra of LiPON films sputter-deposited from Li_3_PO_4_ correspond to N_a_ and N_d_ rather than N_d_ and N_t_. However, this assumes that there is good congruence between the composition of a sputtering target and the films deposited from it. In reality, lithium loss always occurs to some degree during the sputtering of lithium compounds and is dependent not only on the sputter deposition conditions but also on the characteristics of the deposition system used.^53^ Therefore, the chemical compositions of LiPON films vary from study to study. In this study and several previous reports, the LiPON films had similar compositions to LiPO_3_-derived bulk-processed LiPON, which explains the similar molecular structures of these samples despite their fundamentally different processing routes.^21,54−57^ Since their Li/P ratios were well below 2.2, the assignment of the two principal N 1s peaks to N_t_ and N_d_ was justified.

It is nevertheless worth considering why, in the case of the LiPON film, the Li/P ratio decreases from a value of 3 in the sputtering target to 1.60 in the film. Interestingly, the EDX measurements (which are less surface sensitive) imply, by charge balance, that the bulk Li/P ratio could be somewhat higher (∼2). Therefore, the overall lithium deficiency may not be as great as implied by the compositions in Table 1, although it would still result in LiPON of metaphosphate character according to Lacivita et al.^50^ Additional lithium loss may have occurred in our deposition experiments due to the relatively long target-to-substrate distance of our PVD system (∼12 cm), meaning that a large proportion of the lithium (which is much lighter than the other elements in LiPON) was likely deflected before it could reach the substrate.^53^

The ratios of N_t_ to N_d_ and of O_b_ to O_nb_ calculated from component area ratios are presented in Table 1. As expected, given the similar appearance of the spectra, the values of these ratios are similar for the two samples. N_t_ and O_b_ both form linkages between neighboring molecular chains, so the higher concentration of N and Li in the thin film sample may have been responsible for its higher ratio of N_t_ to O_b_. Although P exists in several nonidentical chemical environments due to the various compositions of P(O_*x*_N_*y*_) groups, the similarity of these environments is such that the differences in binding energy cannot be resolved. Thus, the overall emission between 133 and 134 eV is labeled “P(O_*x*_N_*y*_)”. The precise binding energies of the spectral components shown in Figure 3 and their evolution with lithium plating are provided by the plots in Figure S11.

#### Lithiation and Structural Breakdown of the LiPON

**Figure 4:**
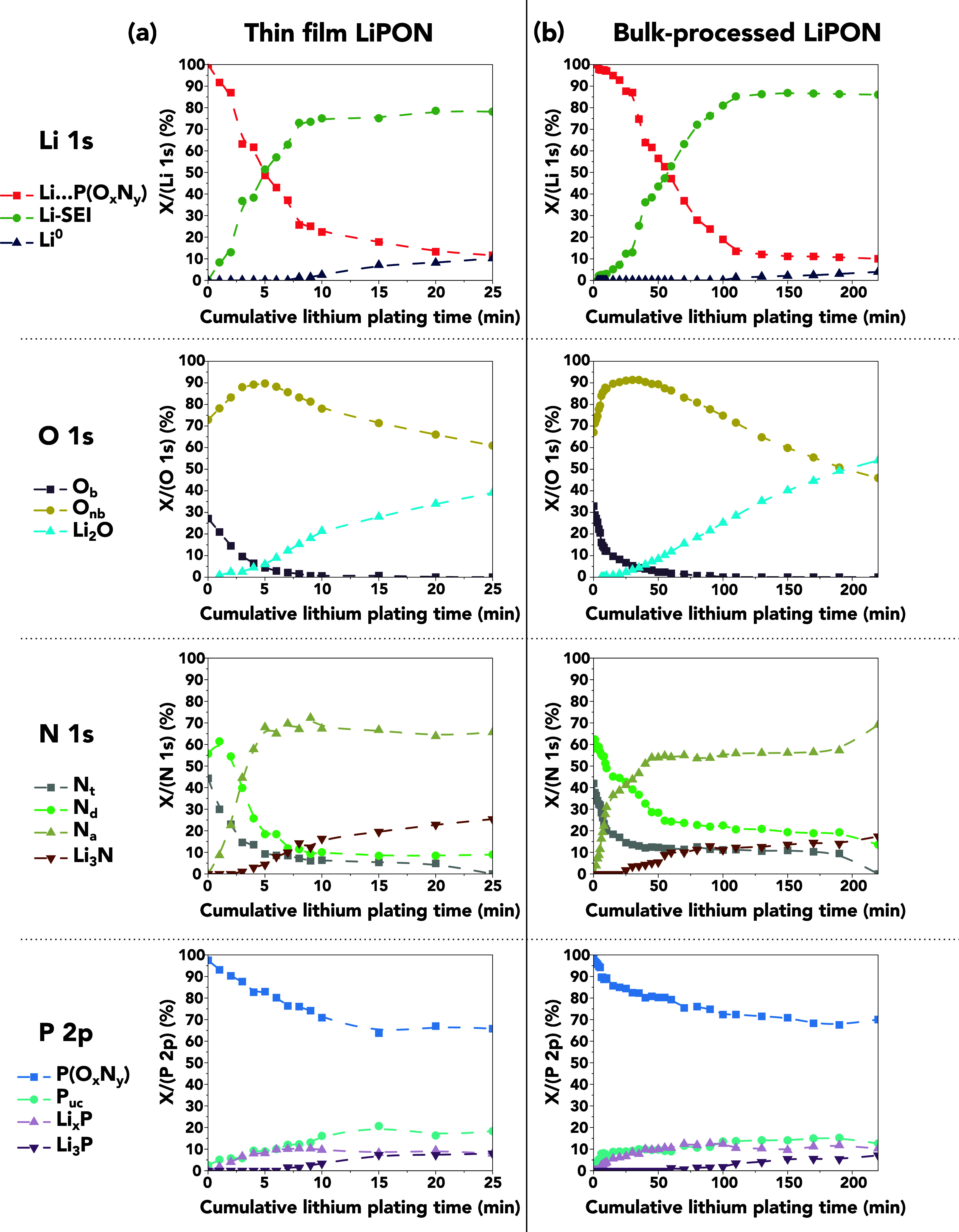
Changes in core-level component (*X*) fractions in each of the core-level spectra in Figure 3 over the cumulative lithium plating time, calculated from the component peak areas. The plots in (a) and (b) correspond to the thin film and bulk-processed LiPON samples, respectively.

We now consider the changes that occurred in the near-surface regions of the two LiPON samples on plating lithium. Figure 3a and b show that new peaks corresponding to SEI compounds appeared in the core-level spectra of both samples after a certain amount of lithium metal had been plated. Structural and chemical changes also occurred within the LiPON. Figure 4 shows the component fractions for each of the core-level spectra in Figure 3 over the cumulative plating time, and the associated binding energies are plotted in Figure S11. The changes in the thin film and bulk-processed LiPON samples followed similar patterns, which should be expected given the similarities in the chemical compositions and initial molecular structures. However, the changes took place over somewhat longer cumulative lithium plating times in the bulk-processed sample, which was primarily due to its greater thickness as discussed later.

The nature of the structural changes within the LiPON samples is shown most clearly by the O 1s and N 1s spectra in Figure 3 and the corresponding plots of the component fractions in Figure 4. As the cumulative plating time increased, the fraction of O_nb_ increased while that of O_b_ decreased. Meanwhile, the fractions of both N_t_ and N_d_ decreased, although the fraction of N_d_ in the thin film briefly increased by over 5 atom % during the initial plating step. A new N 1s component appeared at a lower binding energy, as would be expected for emission from apical nitrogen (N_a_), and its fraction grew at a high rate before leveling off as the fractions of N_d_ and N_t_ reached their baselines.^50^ The disappearance of bridging environments and growth of non-bridging environments suggests that the extended phosphate chain structures within the near-surface regions of the LiPON samples were broken down during lithiation. These structural changes could be described as a transition from metaphosphate to orthophosphate character, consistent with the earlier discussion on the effect of the Li/P ratio on molecular structure. Therefore, it is to be expected that LiPON of orthophosphate character, with an Li/P ratio closer to that of the Li_3_PO_4_ precursor, will have a lower capacity for lithiation and display more minor structural changes when lithium is plated on its surface.

#### Formation and Chemical Evolution of the SEI

The onset of SEI formation was heralded by the emergence of a secondary component in the Li 1s spectra at 54.0–54.6 eV after cumulative plating times of 1 and 4 min for the thin film and bulk-processed LiPON samples, respectively. This component is labeled “Li-SEI” in Figures 3 and 4, and corresponds to Li^+^ in species such as Li_2_O, Li_3_N, and Li_3_P—the binary SEI compounds expected to form based on previous reports.^21,22,24,26^ Since these compounds have very similar binding energies for lithium, their identities were verified from the new components in the other core-level spectra with reference to the attributions made in previous reports. Figure 4 shows that the first binary SEI compound to form was Li_*x*_P, which appeared in the P 2p spectra of the thin film and bulk-processed samples at ∼128.5 eV after 1 and 5 min of lithium plating, respectively. Li_*x*_P is lithium phosphide in which the P has a higher oxidation state than its value in Li_3_P (−3), giving it a higher binding energy. Li_*x*_P has previously been detected in the SEIs of certain sulfide electrolytes.^30,33^

Li_2_O also formed during the first minute of plating on the thin film sample, indicated by the appearance of a peak at ∼528.4 eV in the O 1s spectrum; this peak was not seen until a cumulative plating time of 8 min for the bulk-processed sample. The next binary compound to form was Li_3_N, which appeared in the N 1s spectra of the thin film and bulk-processed samples at ∼395 eV after cumulative plating times of 3 and 25 min, respectively. After respective times of 7 and 60 min, an emission from Li_3_P appeared in the P 2p spectra, centered at a binding energy of ∼126 eV. The delayed appearance of Li_3_P suggests that it formed once some of the Li_*x*_P had fully lithiated to Li_3_P. Further evidence for this was provided by the decline in the binding energy of P in Li_*x*_P toward the value for P in Li_3_P during the formation of the SEI, as shown in Figure S11. After the appearance of Li_3_P, all of the expected binary SEI compounds had formed.

So far, no reference has been made to the “P_uc_” (undercoordinated P) component of the P 2p spectra since it does not correspond to a well-defined LiPON structural unit or SEI compound. In their computational study on the Li|LiPON interface, Sicolo et al. found that the oxidation states of undercoordinated P atoms produced during the breaking of P–N and P–O bonds were reduced to +3, +2, or −2 by reaction with lithium metal.^22^ The growth in P_uc_ fraction from the start of each experiment could be explained by the breaking of P–N and P–O bonds in N_t_, N_d_, and O_b_ units during lithiation of the LiPON. Subsequent reaction between lithium and P–O_nb_ and P–N_a_ could have resulted in the formation of Li_*x*_P as well as Li_2_O and Li_3_N. It should be noted that some P_uc_ may have been present prior to plating any lithium, at least for the bulk-processed sample, as shown more clearly by the magnified P 2p spectra in Figure S12. This P_uc_ could have formed by the cleavage of P–N and P–O bonds during the Ar-ion etching process. Indeed, the P 2p spectra acquired prior to etching (Figure S12) show no emission from P_uc_.

Analysis of the core-level XPS spectra in Figure 3 has revealed that Li_2_O, Li_3_N, and Li_3_P were present in the SEIs formed on both LiPON samples, which is broadly consistent with the findings of previous studies on the Li-LiPON SEI. However, in two of those studies, Li_3_PO_4_ was identified as an SEI phase (either in place of Li_3_P or in addition to it).^21,24^ Identification of Li_3_PO_4_ from the core-level XPS spectra is not straightforward because there is no unique emission from Li_3_PO_4_: its emissions overlap with those from LiPON and contribute to the Li···P(O_*x*_N_*y*_), O_nb_, and P(O_*x*_N_*y*_) components in the Li 1s, O 1s, and P 2p spectra, respectively. Nevertheless, the fraction of the P(O_*x*_N_*y*_) emission attributable to Li_3_PO_4_ can be determined by subtracting all contributions from LiPON (P bonded to N_t_, N_d_, N_a_, and O_b_).

Although the precise molecular structures of the LiPON samples are unknown, the maximum possible content of P attributable to LiPON can be calculated by considering the hypothetical case in which the LiPON consisted of the simplest possible structural units based on N_t_, N_d_, N_a_, and O_b_ (Figure S13). The chemical formulas of these units were used to convert measured atomic fractions of N_t_, N_d_, N_a_, and O_b_ into atomic fractions of P, and the sum was subtracted from the measured atomic fraction of P in P(O_*x*_N_*y*_) to give the minimum possible fraction of P in Li_3_PO_4_. The results of these calculations are plotted in Figure S14, and an example calculation is shown in Table S3.

Early in the plating experiments, the concentrations of Li_3_PO_4_ in the two LiPON samples should have been negligible, which explains why the P concentrations calculated by the above method were initially negative. Nonetheless, the values became positive after ∼7 and ∼20 min of plating before leveling out at ∼1.2 and ∼4 atom % for the thin film and bulk-processed samples, respectively, which means that at least 15 or 50% of the P detected in the samples was present in Li_3_PO_4_ at this time. Therefore, this analysis shows that Li_3_PO_4_ was a major constituent of the SEIs formed in this study.

It is worth considering whether the lithiation of LiPON and subsequent formation of the SEI can be described by chemical reactions consistent with the above observations. From inspection of the hypothetical LiPON fragment in Figure S10, a series of exemplar degradation reactions is proposed in eqs 2–5. Since the detailed molecular properties of the LiPON samples fabricated in this investigation were unknown, these reactions were formulated using the chemical compositions of the isolated structural units shown in Figure S13. It should be noted that intermediate reaction steps involving P_uc_ species are expected from the analysis of the XPS spectra in Figure 3, but to attempt to include these would be to introduce further assumptions that are not required to explain the basic features of lithiation and SEI formation.
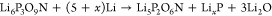
2
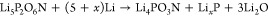
3
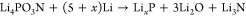
4
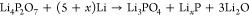
5

Equation 2 shows a pathway for the decomposition of N_t_ units (Li_6_P_3_O_9_N) to N_d_ units (Li_5_P_2_O_6_N), which is consistent with the decline in the N_t_ fraction during the lithium plating experiments (Figure 4). Furthermore, the brief increase in the N_d_/N_t_ ratio at the start of lithium plating on the thin film sample supports the proposed decomposition pathway of N_t_ to N_d_, and the two SEI products formed in this reaction—Li_*x*_P and Li_2_O—were those found to form first in the plating experiments. For both samples, the decline in N_t_ was soon accompanied by a decreasing fraction of N_d_ and an increasing fraction of N_a_, consistent with eq 3, which produces more Li_*x*_P and Li_2_O—the only two SEI products that were detectable when N_a_ first appeared. According to a previous report, P(O_*x*_N_*y*_) units containing N_a_ are unstable against lithium metal and should thus decompose by a reaction such as eq 4.^24^ This reaction produces Li_3_N as well as Li_*x*_P, in agreement with the order of binary SEI compound formation reported earlier. Finally, eq 5, which is based on a decomposition reaction proposed by Schwöbel et al., captures the sharp decline in O_b_ (Li_4_P_2_O_7_) and growth of O_nb_ (Li_3_PO_4_) shown in Figure 4.^21^ Crucially, this reaction provides a pathway for the formation of Li_3_PO_4_.

This analysis of decomposition pathways suggests that the initial lithiation of LiPON and subsequent lithiation of its decomposition products results in the formation of SEI compounds as coproducts. While the same binary compounds may ultimately form regardless of whether the LiPON is of metaphosphate or orthophosphate character, the initial concentrations of N_t_, N_d_, N_a_, and O_b_ could influence the composition and thickness of the SEI. For example, Li_3_N should form earlier in the decomposition of orthophosphate-type LiPON due to the greater initial fraction of N_a_, while more Li_3_PO_4_ could form in the decomposition of metaphosphate-type LiPON owing to its greater initial fraction of O_b_.^50^ The higher initial O_b_/O_nb_ ratio of the bulk-processed LiPON would explain why the fraction of Li_3_PO_4_ in its SEI was greater than that in the SEI of the thin film. Depending on the roles that Li_3_N and Li_3_PO_4_ play in passivating the interface, compositional differences may affect the time taken (and hence the SEI thickness required) for passivation to occur.

#### Passivation of the Li|LiPON Interface and Final Structure of the SEI

As expected, after developing to a certain extent, the Li-LiPON interphase became passivating, impeding further decomposition of the LiPON. This was manifested by the accumulation of unreacted lithium metal on the sample surfaces with continued lithium plating: an Li^0^ (metallic lithium) emission appeared in the Li 1s spectra after 8 and 110 min of plating on the thin film and bulk-processed samples, respectively, and grew in intensity as the cumulative plating time increased (Figure S5). Further evidence for passivation is provided by the plots of core-level component binding energies in Figure S11, most of which ceased to change after the emergence of Li^0^. The exception was the binding energy of the Li-SEI component, which started to decrease for both samples due to a growing O 1s emission from Li_2_O (Figure S5) and diminishing emissions from the other SEI compounds as the lithium metal layer thickened. However, the anomalous increase in Li_2_O was likely due to a reaction between the newly formed lithium metal and trace O_2_ in the XPS chamber, consistent with previous reports.^18,22,30,33,58^

Consideration should now be given to the characteristics of the fully formed SEIs. Since LiPON exists over a broad range of Li^+^ concentrations and undergoes decomposition through a series of lithiation reactions, inward diffusion of lithium from the surface results in the formation of SEIs with nonuniform chemical compositions and structures. It therefore follows that the most and least lithiated species will be found at the lithium metal and LiPON sides of the interphase, respectively, with a lithiation, and hence lithium chemical potential, gradient in between.

The earlier analysis of SEI formation linked the evolution of the core-level XPS spectra to the degree of lithiation through eqs 2–5. Thus, the compositional variations within the SEIs may correspond to a spatial representation of the temporal changes observed during the in situ plating experiments. In that case, the concentrations of O_b_, N_t_, and N_d_ would be highest on the LiPON side of the interphase, species of intermediate lithiation such as P_uc_, Li_*x*_P, N_a_, and Li_3_PO_4_ would be concentrated toward the center, and the fully lithiated compounds (Li_2_O, Li_3_N, and Li_3_P) would be predominant on the lithium metal side. Although XPS does not provide a complete picture of the final SEI structure, some key properties of the SEIs were determined from the core-level spectra acquired after passivation. This was facilitated by a combination of three factors: (1) the “frozen” SEI and LiPON structures after passivation, (2) the resulting accumulation of lithium metal during further plating, and (3) the shallow probing depth of XPS. Since the chemical changes within the LiPON samples and their SEIs were negligible after passivation, the continuing evolution of the core-level spectra reflected the changing sampling location of the XPS probe as the accumulated lithium thickened. The fractions of most of the core-level components corresponding to SEI species became constant or grew slightly after passivation (Figure 4), reflecting the increasing proportion of the probing depth occupied by the SEI compared to that occupied by the LiPON.

The decreases in Li···P(O_*x*_N_*y*_) and O_nb_ fractions were due to the strong growth in Li 1s and O 1s emissions from Li^0^ and Li_2_O, respectively. The only other component fractions seen to decrease markedly for both samples at some point after passivation were those of O_b_ and N_t_. There is some uncertainty as to whether these components should be considered part of the SEIs. Such bonding configurations should only be present during the initial stages of lithiation, which can be seen as a transition within the LiPON from metaphosphate to orthophosphate character. Indeed, these components were the only ones to die out completely during the lithium plating after passivation, confirming that the species from which they originated were concentrated close to the interface with the LiPON. Since the emissions from the O_b_ became negligible at approximately the time that the SEIs stopped growing, the SEI thicknesses were likely on the order of the XPS probing depth, which is determined by the inelastic mean free path (λ_IMFP_) of the photoelectrons. For the elements in LiPON, the kinetic energies of ejected photoelectrons are higher than 950 eV, resulting in a λ_IMFP_ ranging from 38 to 54 Å in lithium metal.^59^ The effective probing depth for XPS is approximately 3λ_IMFP_, so the probing depth in lithium metal was about 11–16 nm. This provides an upper limit in the estimation of SEI thickness: P, O, and N are heavier than Li, so the λ_IMFP_ values for LiPON and its SEI would have been lower.

Therefore, the Li-LiPON SEI thickness determined in our investigation is in reasonable agreement with the values (∼5 nm) reported by Westover et al. and Browning et al. in their electroanalytical and neutron reflectometry studies.^27,28^ By contrast, Cheng et al. and Hood et al. reported significantly thicker SEIs of 76 and ∼60 nm, respectively, based on their TEM characterization.^24,25^ These significantly greater SEI thicknesses could have been due to the fact that electrochemical plating of lithium was not performed in the TEM studies, which may have resulted in a lower rate of passivation than observed in our in situ lithium plating XPS study and the electroanalytical and neutron reflectometry studies mentioned above. However, there is a somewhat higher uncertainty associated with measurements of SEI thickness made using XPS than with those made by direct imaging techniques such as TEM. Since the emission from O_b_ in the bulk-processed sample died out slightly earlier with respect to the appearance of Li^0^ than that from O_b_ in the thin film, the SEI formed on the bulk-processed sample may have been thicker. This is consistent with the lower lithium content of the bulk-processed sample (Table 1) and its consequently higher capacity for lithiation.

The N 1s spectral components arising from the primary decomposition products of LiPON—N_d_ and N_a_—should now be considered. These bonding configurations have not previously been associated with the Li-LiPON SEI, yet examination of Figure 4 suggests that they were both present since their fractions were nonzero after both O_b_ and N_t_ had vanished. Nevertheless, the N_d_ fraction in the bulk-processed sample declined sharply between cumulative plating times of 190 and 220 min. This suggests that species containing N_d_ bonding configurations were concentrated near the interface with LiPON, as expected from the earlier analysis. By contrast, the N_a_ fraction remained constant for the thin film sample and grew sharply for the bulk-processed sample at the end of lithium plating, and Figure S5 shows that clear N 1s emissions from N_a_ were still visible for both samples. This indicates that the N_a_ was concentrated further away from the LiPON side of the interphase than N_d_, which was also predicted from the earlier analysis of LiPON decomposition.

The plateauing of the P 2p emission from P(O_*x*_N_*y*_) provides further evidence that N_a_ and Li_3_PO_4_ were present in the bulk of each SEI, although the distribution of Li_3_PO_4_ relative to N_a_ cannot be determined. As for N_a_, the core-level emission fractions from P_uc_ and Li_*x*_P initially rose and then leveled off after passivation, suggesting that they were also concentrated away from the LiPON side of each SEI. The core-level emission fractions from Li_3_P and Li_3_N continued to rise during lithium accumulation on the samples after passivation, consistent with the expected predominance of these species at the lithium metal side of each SEI.

**Figure 5:**
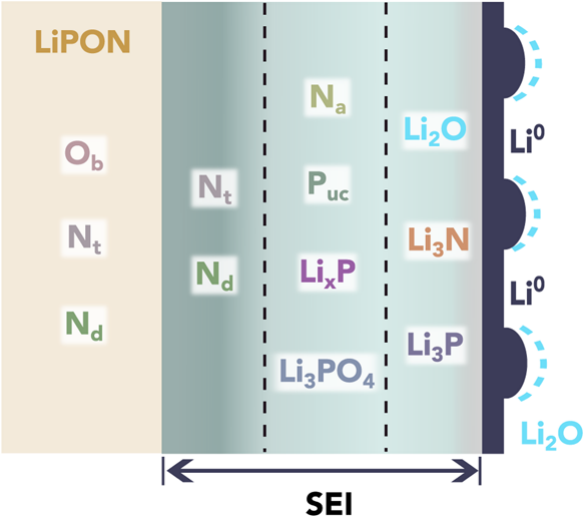
Schematic diagram of the Li-LiPON SEI structure determined from the results of the in situ lithium plating XPS experiments. The dashed lines between the layers of the SEI structure indicate that these are not “hard” boundaries and some degree of intermixing is expected. Lithium metal is depicted as an inhomogeneous layer, since electrodeposited lithium rarely grows in a uniform manner.^58,60^ The layer thicknesses are not drawn to scale.

Our proposed distribution of species within the Li-LiPON SEI, shown schematically in Figure 5, is broadly consistent with the graded SEI structure reported by Sicolo et al. in their XPS study, although additional SEI species including P_uc_, Li_*x*_P, and N_a_ are reported here, and our results indicate the presence of an additional layer rich in N_d_ on the LiPON side of the SEI.^22^ However, the TEM studies of Cheng et al. and Hood et al., and the computational study of Wang et al., conclude that Li_2_O is the SEI compound closest to the interface with lithium metal.^24−26^ This may contradict our finding that the final SEI compound to form before passivation was Li_3_P, which suggests that Li_3_P was in direct contact with the lithium metal after passivation, along with Li_2_O and Li_3_N.

Interestingly, two recent studies found that Li_3_P destabilizes the interphase formed by a sulfide solid electrolyte in contact with lithium metal. Liang et al. showed that Li|LGPS formed a mixed conducting interphase due to the continuous electron-conducting pathway provided by the percolation of Li_3_P, which has *a* relatively high electronic conductivity (∼10^–4^ S cm^–1^).^61−63^ Burton et al. found that the low potential required for the oxidation of Li_3_P to Li_*x*_P (0.87 V vs Li^+^/Li) in the Li–Li_6_PS_5_Cl interphase resulted in additional reduction of the solid electrolyte, since Li_6_PS_5_Cl has a theoretical reduction potential of 1.71 V vs Li^+^/Li.^23,64^ Crucially, the oxidation of Li_*x*_P, which forms a percolating network, can also occur below this reduction potential, meaning that while lithium metal was present to “feed” the constant redox of the phosphide species, continuous, diffusion-limited growth of the Li–Li_6_PS_5_Cl interphase ensued. There are two properties of the Li-LiPON SEI that prevent the facilitation of continuous interphase growth by the most highly lithiated SEI species (Li_2_O, Li_3_N, and Li_3_P). First, the reduction potential computed for LiPON (0.68 V) by Zhu et al.^23^ is below the oxidation potentials of these binary compounds, and second, the graded structure of the Li-LiPON SEI ensures that the most reduced species are not in physical or electrical contact with the LiPON layer: a percolating network of these species does not exist.

#### Effect of Sample Thickness

We have shown that SEI formation on the thin film and bulk-processed LiPON samples was similar in most respects, the main difference being the cumulative plating times over which the chemical and structural changes occurred. Since the SEIs had similar thicknesses, similar plated capacities of lithium should have been required to form them. Therefore, it is important to consider whether the actual current densities applied to the samples were equal to the value calculated from the electron flood gun current and exposed sample area.

The equivalent circuit diagrams used to fit the EIS results in Figures S3 and S4 should be valid for modeling the electrical response of the samples during in situ lithium plating, although the interfaces with the “electrodes” were essentially non-blocking in these experiments, so the interfacial capacitances will be neglected. Further, the interfacial resistance, *R*, will be considered part of the ionic resistance of the sample, *R*_i_, to simplify the following analysis.

In theory, on applying a constant current, *I*_gun_, of 30 μA, the potential difference, *V*_sample_, across *R*_i_ will rise to a value given by *V*_sample_ = *I*_gun_*R*_i_. This cannot occur instantaneously because some of the applied current will flow into the branch of the circuit containing the geometric capacitance, CPE_geom_. The current flowing into this capacitance will decrease as the potential difference increases and will stop once it reaches *V*_sample_. The physical manifestation of this is the accumulation of electrons on the LiPON surface until the potential difference required to drive the applied current (30 μA) through *R*_i_ has been reached and *I*_sample_ = *I*_gun_.

This analysis raises the possibility that the capacitance of the bulk-processed sample could have accounted for its seemingly lower average value of *I*_sample_ during each plating step. However, examination of the EIS results shows that it could not have: the CPE_geom_ capacitances calculated by fitting the room temperature Nyquist plots in Figures S3 and S4 were ∼1 × 10^–9^ and ∼5 × 10^–10^ F for the thin film and bulk-processed samples, respectively. Not only are these values very small, but also the capacitance of the thin film sample was higher than that of the bulk-processed sample. The capacitance of the bulk-processed sample would likely have been even lower in practice due to the greater thickness of the sample used in the in situ plating experiment.

We then considered the possibility that the true steady-state value of *I*_sample_ was lower for the bulk-processed sample than for the thin film, despite setting *I*_gun_ = 30 μA. This situation would have occurred if electrons from the flood gun were deflected by the electrons on the sample surface. To determine whether this was likely, we calculated the *V*_sample_ values for *I*_sample_ = 30 μA. The values of *R*_i_ measured at room temperature by EIS were ∼5000 Ω for the thin film sample and ∼7 × 10^7^ Ω for the bulk-processed sample. However, in those experiments the diameter of the top electrical contact was 2 mm, whereas in the in situ plating experiments the diameter of the current path was 5 mm and the bulk-processed sample was twice as thick. Thus, the corrected *R*_i_ values were ∼800 and ∼2 × 10^7^ Ω. The corresponding values of *V*_sample_ were ∼0.024 and ∼600 V, respectively. Since the accelerating voltage of the electron flood gun, *V*_gun_, was only ∼1 V, this was the maximum potential drop that could have occurred through each sample. Thus, the value of *V*_sample_ calculated for the bulk-processed sample could not have been reached; the actual current through the sample would have been no more than  (0.05 μA) assuming a maximum possible value of 1 V for *V*_sample_. The additional current provided by the flood gun (*I*_gun_ – *I*_sample_) must have been deflected to ground by the surface charge on the bulk-processed sample. By contrast, the value of *V*_sample_ calculated for the thin film sample is significantly lower than *V*_gun_, so it is likely that *I*_sample_ was much closer to *I*_gun_ for this sample. Nevertheless, some deflection of the incident electrons could still have occurred.

To determine whether deflection of the incident electron beam can occur even when *V*_sample_ is well below *V*_gun_, as was the case for the thin film sample, we performed an additional in situ lithium plating XPS experiment on an “ultrathin” LiPON film with a thickness of ∼0.01 μm. The acquired core-level spectra, shown in Figure S15, are very similar in appearance to those of the thin film and bulk-processed samples in Figure 3, suggesting that the SEI composition, structure, and formation processes were similar for all three samples. Furthermore, the thickness of the SEI formed on the ∼0.01 μm sample was also on the order of the XPS probing depth, evidenced by the O_b_ emission becoming negligible when the Li^0^ emission appeared. The fact that Li^0^ was first detected at a cumulative plating time of 3.5 min means that the SEI formed in approximately half the time required for the ∼0.6 μm film. We were unable to measure the ionic conductivity of the ∼0.01 μm thick LiPON owing to its thinness and consequent susceptibility to short-circuiting, but if we assume the conductivity was the same as that of the ∼0.6 μm film, *R*_i_ would have been ∼10 Ω, giving a *V*_sample_ of ∼3 × 10^–4^ V for *I*_sample_ = 30 μA. Thus, a dependence of the lithium plating rate on sample thickness was evident even at thicknesses for which . This suggests that, for the condition *I*_sample_ = *I*_gun_ to be met, the condition *V*_sample_ ≪ *V*_gun_ must apply, which requires very thin samples in the case of LiPON. If we assume that *V*_sample_ ≪ *V*_gun_ was satisfied for the ultrathin sample, multiplying the nominal applied current density (0.15 mA cm^–2^) by the time to passivation (∼0.06 h) gives the approximate areal capacity of lithium plated prior to passivation (∼8.75 μAh cm^–2^). This is somewhat larger than the capacity of lithium consumed in SEI formation reported by Westover et al. (∼1 μAh cm^–2^). Nevertheless, it is important to consider that some capacity of lithium beyond that required for passivation would have been plated prior to the emergence of a detectable Li^0^ photoelectron signal. This analysis provides further evidence that the thicknesses of the Li-LiPON SEIs formed in our investigation were comparable to those reported by Westover et al. and Browning et al.^27,28^

Despite the advantages of the in situ lithium plating technique over other in situ lithium deposition XPS techniques, the fact that *I*_sample_ = *I*_gun_ is not necessarily satisfied is a clear limitation that must be considered in future studies if the applied current density is considered important. Interestingly, our results suggest that the properties of the fully formed Li-LiPON SEI do not depend strongly on the applied current density, at least for values below 0.15 mA cm^–2^, since the SEIs formed had comparable chemical and structural properties and grew to similar thicknesses despite the broad range of applied current density.

## Conclusions

We have reported the use of an in situ lithium plating XPS technique to study the formation and properties of the Li-LiPON SEI. XPS spectra acquired prior to lithium plating revealed that the thin film and bulk-processed LiPON samples fabricated in this investigation had similar metaphosphate glass structures, which was unexpected considering the differences in precursor composition and fabrication route. We attributed the structural similarities to the comparable [Li]/[P] ratios of thin film and bulk-processed samples—a result of lithium loss during sputter deposition.

On plating lithium, we found that SEI formation proceeded in a similar manner on all of the samples. The LiPON lithiated progressively through a series of reactions that resulted in a graded SEI structure with the most lithiated species (Li_2_O, Li_3_N, and Li_3_P) closest to the interface with lithium metal and the least lithiated species (N_t_ and N_d_) closest to the interface with LiPON. Species of intermediate lithiation were concentrated toward the center of the SEI. These species included P_uc_, Li_*x*_P, and N_a_. Another of these species was Li_3_PO_4_, which we confirmed was present in the SEI in addition to Li_3_P. Our analysis indicated that the concentration of Li_3_PO_4_ relative to Li_3_P would have been lower if the LiPON had been of orthophosphate character, since a significant amount of Li_3_PO_4_ is expected to form by the breaking of the O_b_ structural units. The final thicknesses of the SEIs were also similar for the three samples and were on the order of the XPS probing depth (<16 nm). This was significantly thinner than reported in previous TEM-based studies (60–80 nm), but in reasonable agreement with the values reported in two studies which, like our study, used electrochemical lithium plating to form the Li|LiPON interface (∼5 nm). The discrepancy may have been due to a higher rate of passivation achieved by the electrochemical driving force associated with lithium plating.

The key difference between the samples was the timescale over which changes occurred during lithium plating: the cumulative plating time required for passivation was found to increase with sample thickness for a constant electron flood gun current of 30 μA. This revealed an important limitation of the in situ lithium plating XPS technique when applied to LiPON: the low accelerating voltage of standard electron flood guns and high impedance of typical LiPON samples can result in an applied current density that is significantly lower than the nominal value. The actual current density decreases sharply with increasing sample thickness due to the growing fraction of the electron beam current deflected to ground by the surface charge. Our awareness of this phenomenon allows us to conclude that the properties of the Li-LiPON SEI do not have a strong dependence on current density, at least up to 0.15 mA cm^–2^. In future investigations that employ this technique, calculations should be performed to determine whether deflection of the applied electron beam is likely to occur for a particular experimental setup. The use of electron flood guns with higher accelerating voltages could also help avoid the aforementioned issues.

Our results show that the ability of LiPON to lithiate progressively and form a graded interphase separating highly lithiated, electronically conducting species from the LiPON layer, as well as the low theoretical reduction potential of LiPON, is responsible for the observed electrochemical stability of the Li|LiPON interface. Glassy materials systems should in general accommodate a greater range of Li^+^ concentrations than crystalline compounds, which have to satisfy stringent requirements with respect to lattice symmetry, strain, and local charge interactions, and may therefore have a greater propensity to form graded interphases. Another expected benefit of glassy solid electrolytes is an increased resistance to lithium dendrite formation and growth due to the absence of crystallographic defects. Therefore, the results of this investigation justify renewed interest in glassy solid electrolytes. The use of sputter deposition could expedite the discovery of new glassy solid electrolyte materials, since the development of bulk synthesis routes is often not straightforward.

## Supporting Information Available

Additional tables, figures, and notes, including RSF values, photographs, SEM micrographs, EIS results, EDX compositions, structural formulas, and additional XPS spectra and analysis (PDF)
