# Supplementary material for: Origin of Stability in the Solid Electrolyte Interphase formed between Lithium and Lithium Phosphorus Oxynitride
Source: Chem Mater. 2025 Apr 25;37(9):3504–18. doi: 10.1021/acs.chemmater.5c00483 (PMC12080378; doi:10.1021/acs.chemmater.5c00483)
Supplement: Supplementary file 1 — cm5c00483_si_001.pdf [file cm5c00483_si_001.pdf]

Origin of Stability in the Solid Electrolyte Interphase formed between Lithium and Lithium Phosphorus Oxynitride SUPPORTING INFORMATION

Stephen J. Turrell[†1,2], Yi Liang[†1], Tiancheng Cai[1,2], Ben Jagger[1], and Mauro Pasta[*1,2]

[1]Department of Materials, University of Oxford, Parks Road, Oxford OX1 3PH, United Kingdom

[2]The Faraday Institution, Quad One, Becquerel Avenue, Harwell Campus, Didcot, OX11 0RA, United Kingdom

[†]Author contribution: Stephen J Turrell and Yi Liang contributed equally to this work

[*]Corresponding author: mauro.pasta@materials.ox.ac.uk

Table S1: RSF×T(E) values (the products of the relative sensitivity factor and transmission function) calculated for the elements in LiPON by performing XPS measurements on reference samples in our instrument at a pass energy of 55 eV.

Li 1s	O 1s	P 2p	N 1 s
1.0±0.0	33.5±2.6	25.3±1.8	17.3±3.5

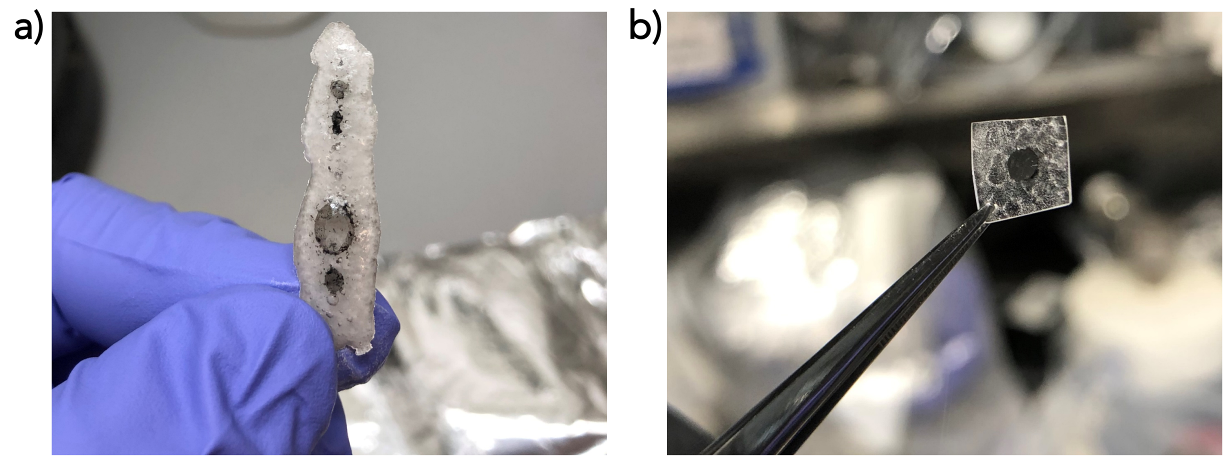

Figure S1: Photographs of the bulk-processed LiPON a) after removing from the alumina boat at the end of the synthesis process and b) after grinding to form a parallel-sided sample, polishing on 1 µm diamond lapping film and depositing 2 mm diameter nickel electrical contacts for impedance measurements.

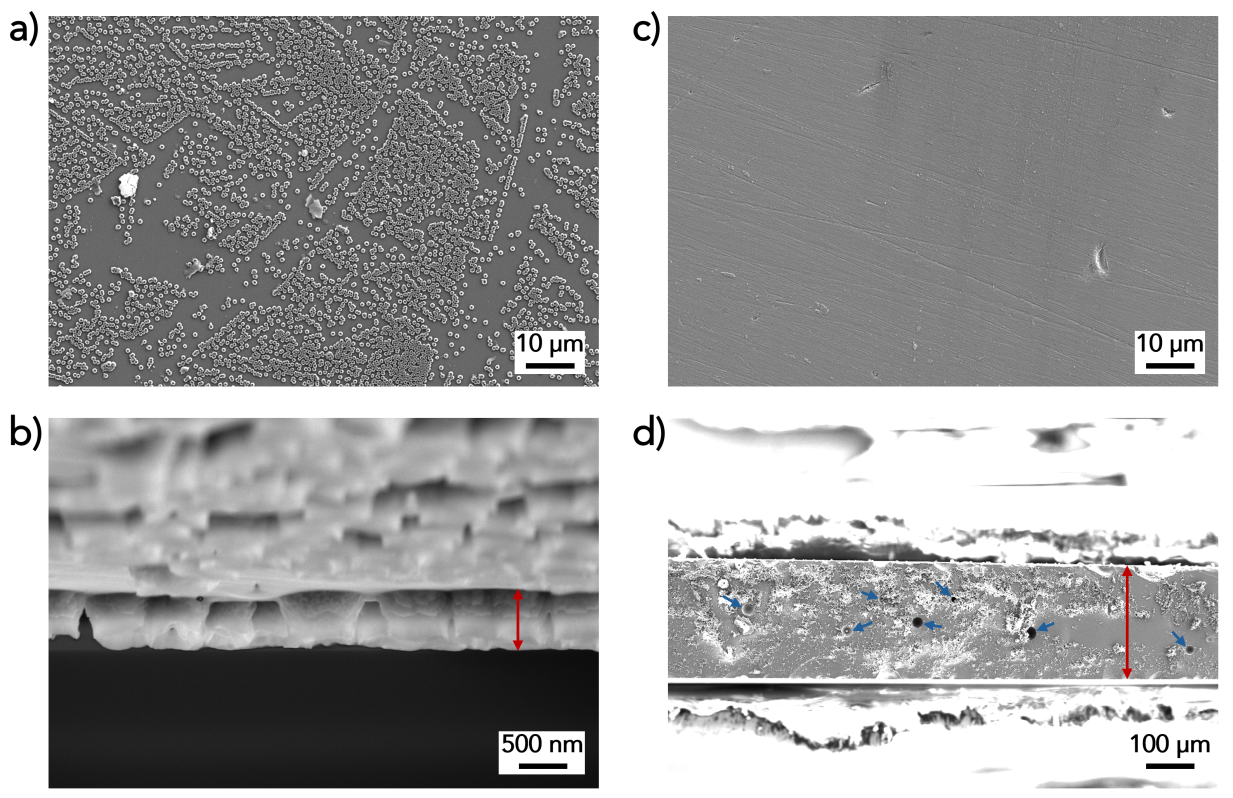

Figure S2: Secondary electron SEM micrographs of a) and b) a LiPON film sputter deposited on undoped silicon at 50 W for 6 hours, and c) and d) a bulk-processed LiPON sample. The cross-sections shown in b) and d) are fracture surfaces and the red arrows indicate the spans of the LiPON layers. The blue arrows highlight the locations of distinct bubbles which intersect the fracture surface.

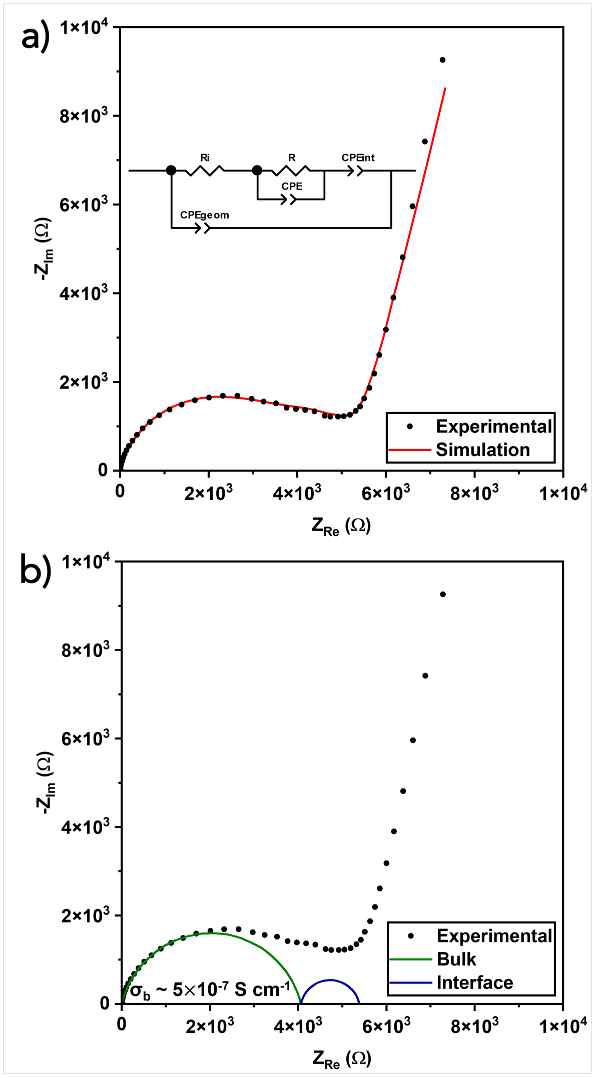

Figure S3: Nyquist plots from electrochemical impedance spectroscopy (EIS) measurements on a ∼0.6 µm thick LiPON film at room temperature. For clarity, measurements at frequencies below 16.3 Hz have not been plotted. The equivalent circuit used to simulate the Nyquist plot consists of resistances (R) and constant phase elements (CPE) and is shown in a) along with the simulation result. ‘R_i’ corresponds to the ionic resistance of the LiPON electrolyte, ‘R’ to the resistance of an interfacial layer, ‘CPEgeom’ to the geometric capacitance, ‘CPE’ to the interfacial layer capacitance and ‘CPEint’ to the blocking electrode interfacial capacitance. The contributions to the sample impedance are plotted as two semicircles in b) to enable the magnitudes of the bulk and interfacial layer impedances to be compared. The ionic conductivity of the LiPON (σ_b, calculated from R_i) was $\sim 5\times10^{-7}$ S cm^{-1}.

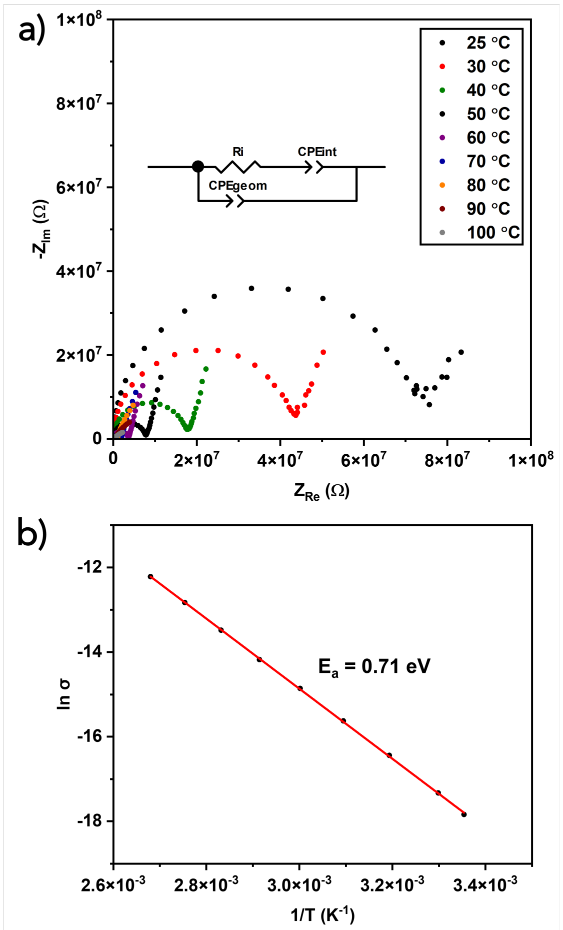

Figure S4: Results of electrochemical impedance spectroscopy (EIS) measurements on a ∼400 µm thick bulk-processed LiPON sample at temperatures between 25 °C and 100 °C. The Nyquist plots recorded at each temperature are shown in a) along with the equivalent circuit used for simulation, which consists of a resistance (R) and constant phase elements (CPE). 'R_i' corresponds to the ionic resistance of the LiPON electrolyte, 'CPE_{geom}' to the geometric capacitance and 'CPE_{int}' to the blocking electrode interfacial capacitance. The values of R_i determined from simulations of the Nyquist plots were used to calculate the ionic conductivity of the LiPON at each temperature, and these values were used to construct the Arrhenius plot in b). Least squares fitting was used to fit a linear trendline to the data, and the activation energy for Li^+ conduction was calculated from the gradient.

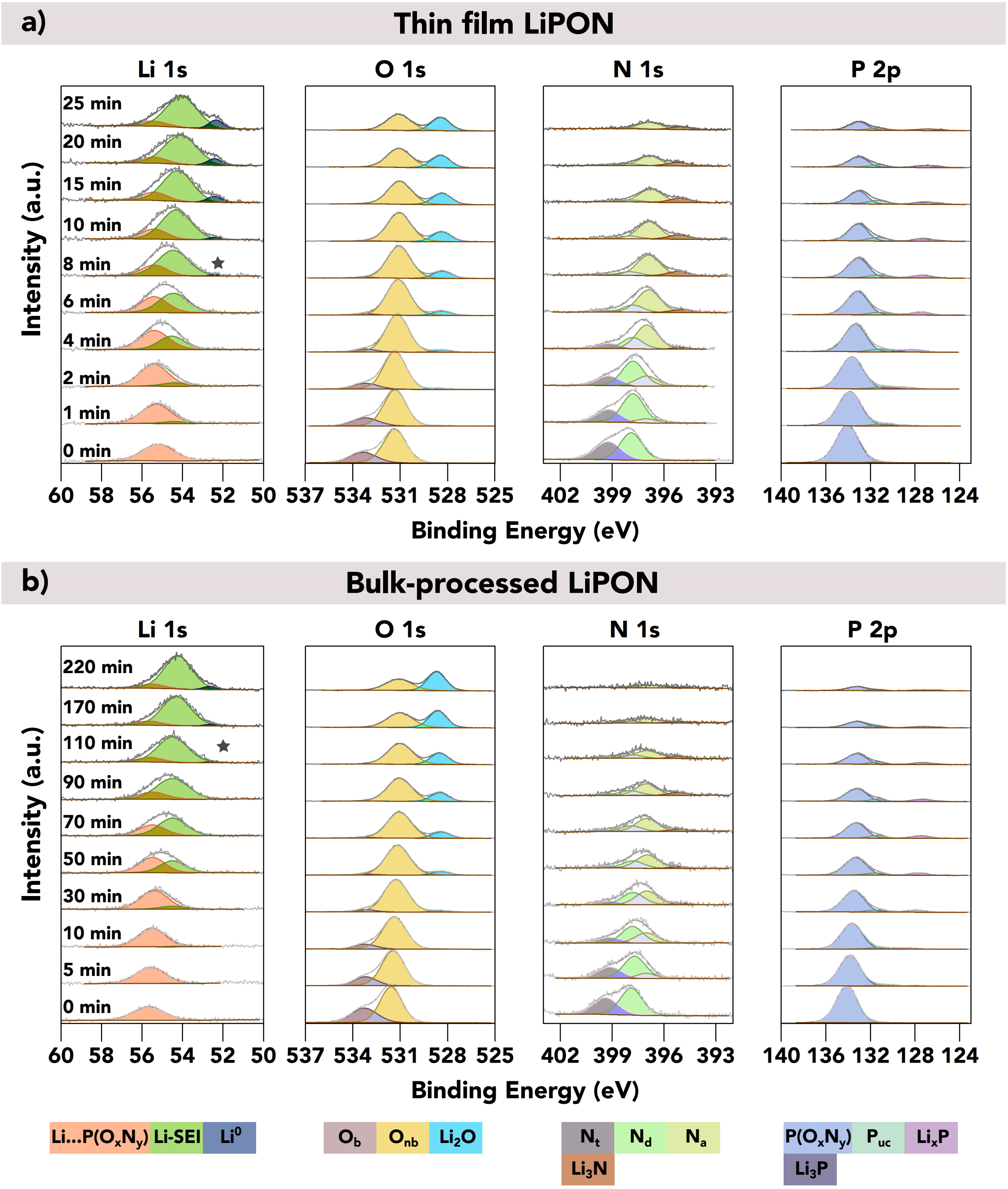

Figure S5: Unnormalized version of Figure 3. Time series of XPS core-level spectra acquired during the *in situ* lithium plating experiments performed on a) a ~0.6 µm thick LiPON film sample and b) an ~800 µm thick bulk-processed LiPON sample. Acquired spectra (grey) are shown along with linear combination fitting results. The cumulative lithium plating time (electron beam exposure time) is indicated in the first column. For clarity, spectra acquired at intermediate plating times are not displayed. Individual spectral components are coloured, and a key is provided below each group of spectra. O_b, O_{nb}, N_t, N_d, N_a and P_{uc} are bridging oxygen, non-bridging oxygen, triple-bridging nitrogen, double-bridging nitrogen, apical (non-bridging) nitrogen and undercoordinated phosphorus, respectively. The appearance of the Li^0 (lithium metal) peak is highlighted with a star. As these spectra have not been normalized, the intensities of emissions from the LiPON – and later from the SEI layer – decreased as the lithium metal layer thickened with increasing cumulative plating time.

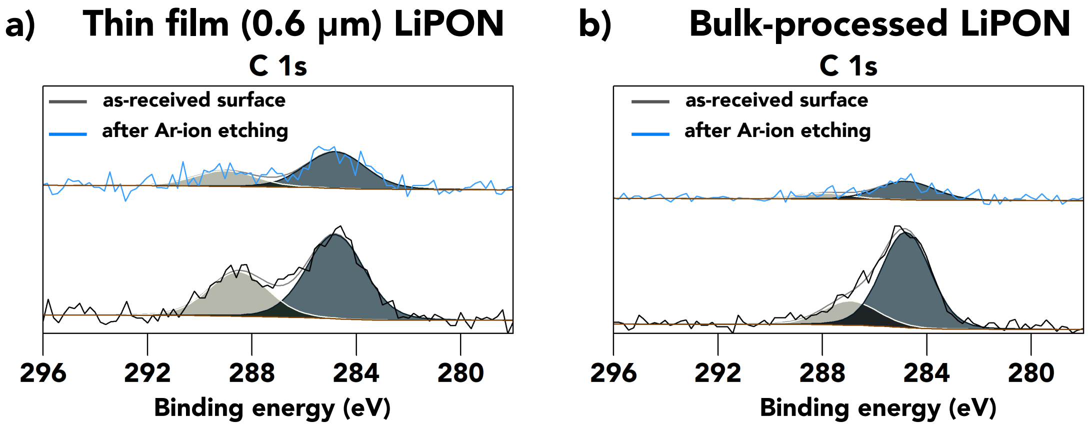

Figure S6: Non-normalized C 1s XPS spectra extracted from survey scans for a) the ∼0.6 µm thick LiPON film sample and b) the ∼800 µm thick bulk-processed LiPON sample before and after Ar-ion etching.

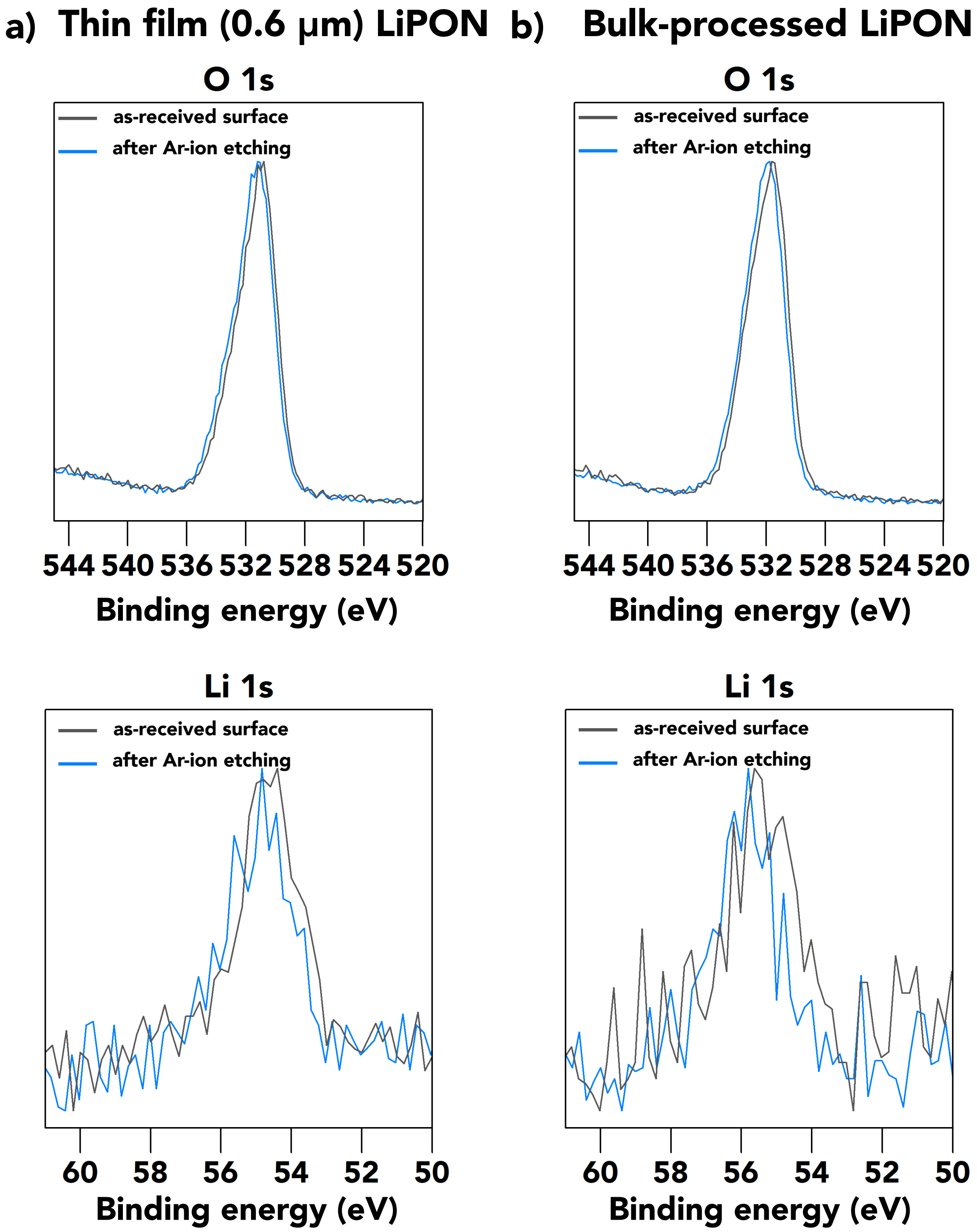

Figure S7: Normalized O 1s and Li 1 s XPS spectra extracted from survey scans for a) the ∼0.6 µm thick LiPON film sample and b) the ∼800 µm thick bulk-processed LiPON sample before and after Ar-ion etching.

Supporting Note 1

A minor peak corresponding to O-N=O (NO_2^-) is present in the N 1s spectrum of the bulk-processed LiPON sample acquired after the initial Ar-ion etching (Figure S8 b)). This peak was not present before Ar-ion etching and it was not seen in the N 1s spectra of the thin film sample acquired before or after Ar-ion etching. The Ar-ion etching may have resulted in the cleavage of P-O and P-N bonds on the surface of the bulk-processed sample and the subsequent reaction of liberated O and N to form NO_2^-. It is not clear why a similar process did not occur on the thin film sample unless the surface properties were slightly different from those of the bulk. Since NO_2^- is a minor surface phase and not part of the LiPON structure it would not have had a significant effect on SEI formation. Therefore, it has been excluded from the spectra in Figure 3 and subsequent analyses, such as the calculations of chemical composition (Table 1) and N 1s component fractions (Figure 4 b)). However, the removal of some of the nitrogen from the LiPON surface to form this phase means that the bulk-processed LiPON composition in Table 1 is slightly deficient in nitrogen and may not be representative of the entire sample. When the contribution from the NO_2^- peak is included, the composition of the bulk-processed sample is $Li_{1.30}PO_{2.65}N_{0.29}$.

Table S2: Chemical compositions determined from energy dispersive X-ray spectroscopy measurements on a ~0.6 µm thick LiPON film sputter deposited on undoped silicon and an ~800 µm thick bulk-processed LiPON sample. The lithium content is not provided because the X-ray signal from lithium cannot be detected using standard EDX detectors.

	Thin film LiPON	Bulk-processed LiPON
Chemical composition	$Li_xPO_{2.57}N_{0.62}$	$Li_xPO_{2.58}N_{0.52}$

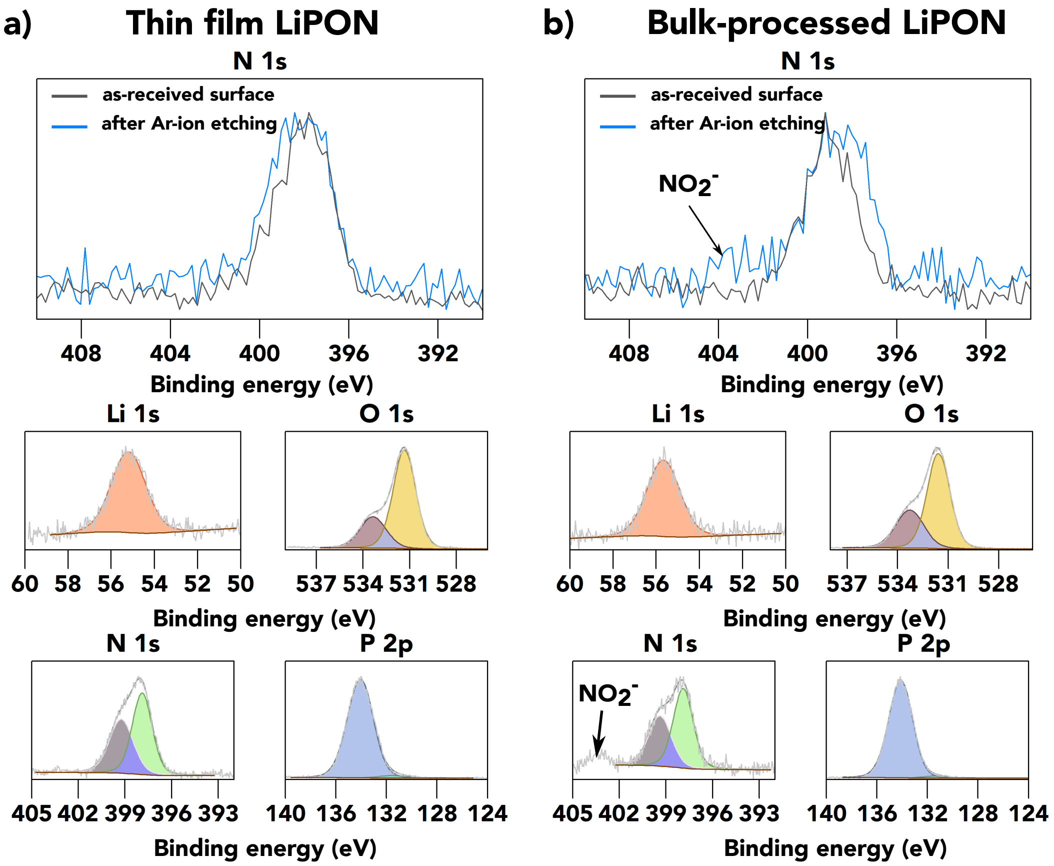

Figure S8: Normalized N 1s XPS spectra in the binding energy range of 409 eV to 391 eV extracted from survey scans for a) the ∼0.6 µm thick LiPON film sample and b) the ∼800 µm thick bulk-processed LiPON sample before and after Ar-ion etching, with the high-resolution core-level spectra after Ar-ion etching included for both samples.

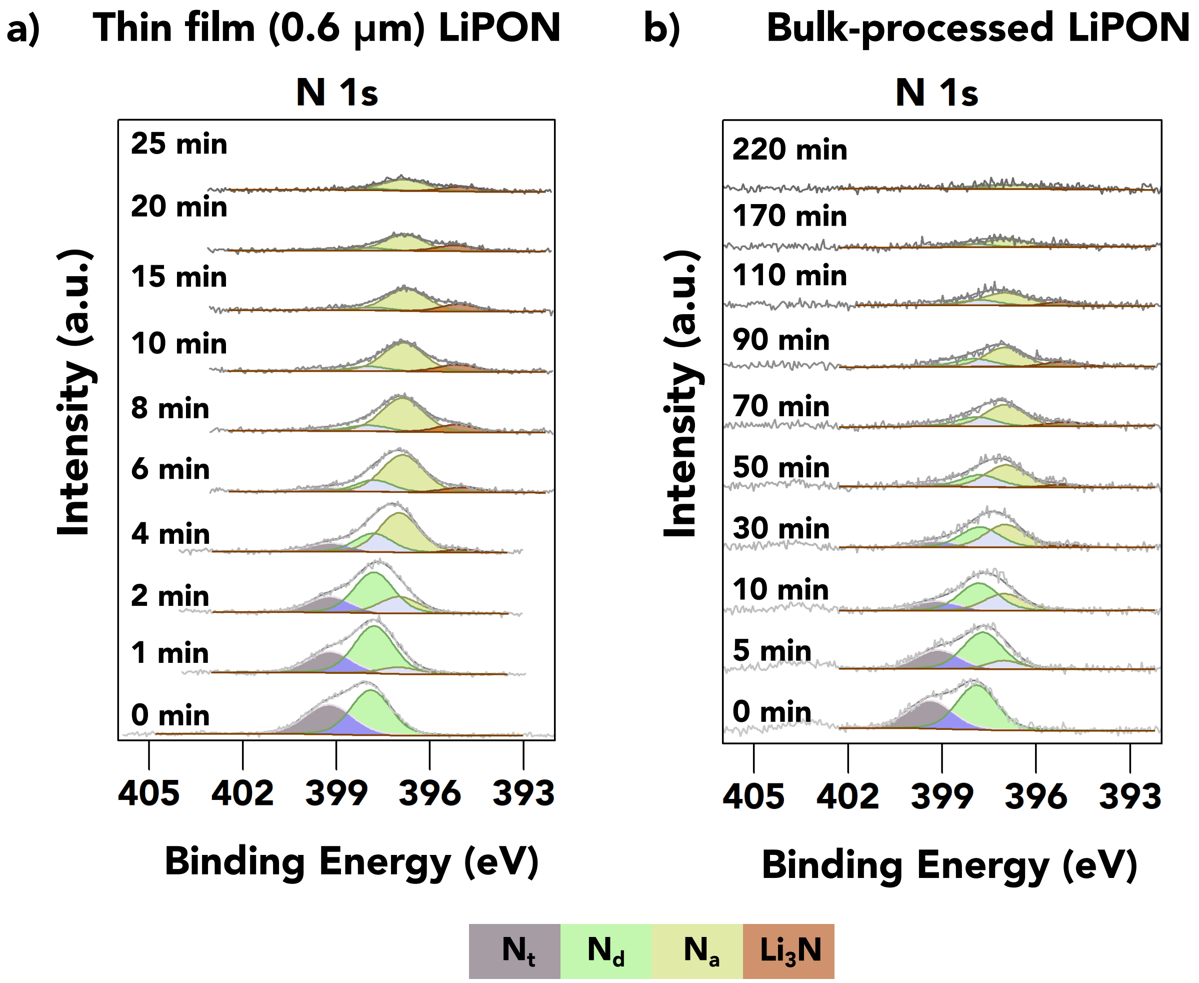

Figure S9: Time series of unnormalized, full-range XPS N 1s spectra acquired during the *in situ* lithium plating experiments performed on a) a ∼0.6 μm thick LiPON film sample and b) an ∼800 μm thick bulk-processed LiPON sample. Acquired spectra (grey) are shown along with linear combination fitting results. The cumulative lithium plating time (electron beam exposure time) is indicated in the first column. For clarity, spectra acquired at intermediate plating times are not displayed. Individual spectral components are coloured, and a key is provided below the spectra. N_t, N_d and N_a are triple-bridging nitrogen, double-bridging nitrogen and apical (non-bridging) nitrogen, respectively. As these spectra have not been normalized, the intensities of emissions from the LiPON – and later from the SEI layer – decreased as the lithium metal layer thickened with increasing cumulative plating time.

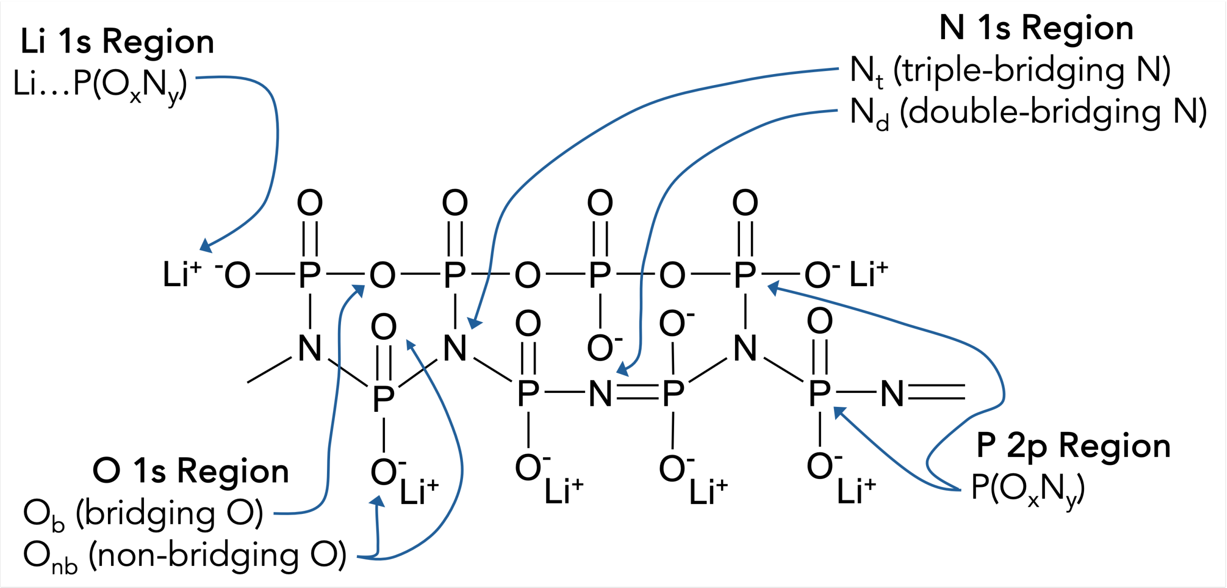

Figure S10: Structural formula of a hypothetical LiPON fragment, with examples of the different chemical environments distinguishable in XPS spectra highlighted. The relative proportions of the different chemical environments in this fragment are not necessarily representative of the LiPON compositions reported in this study. Drawn with reference to [1]

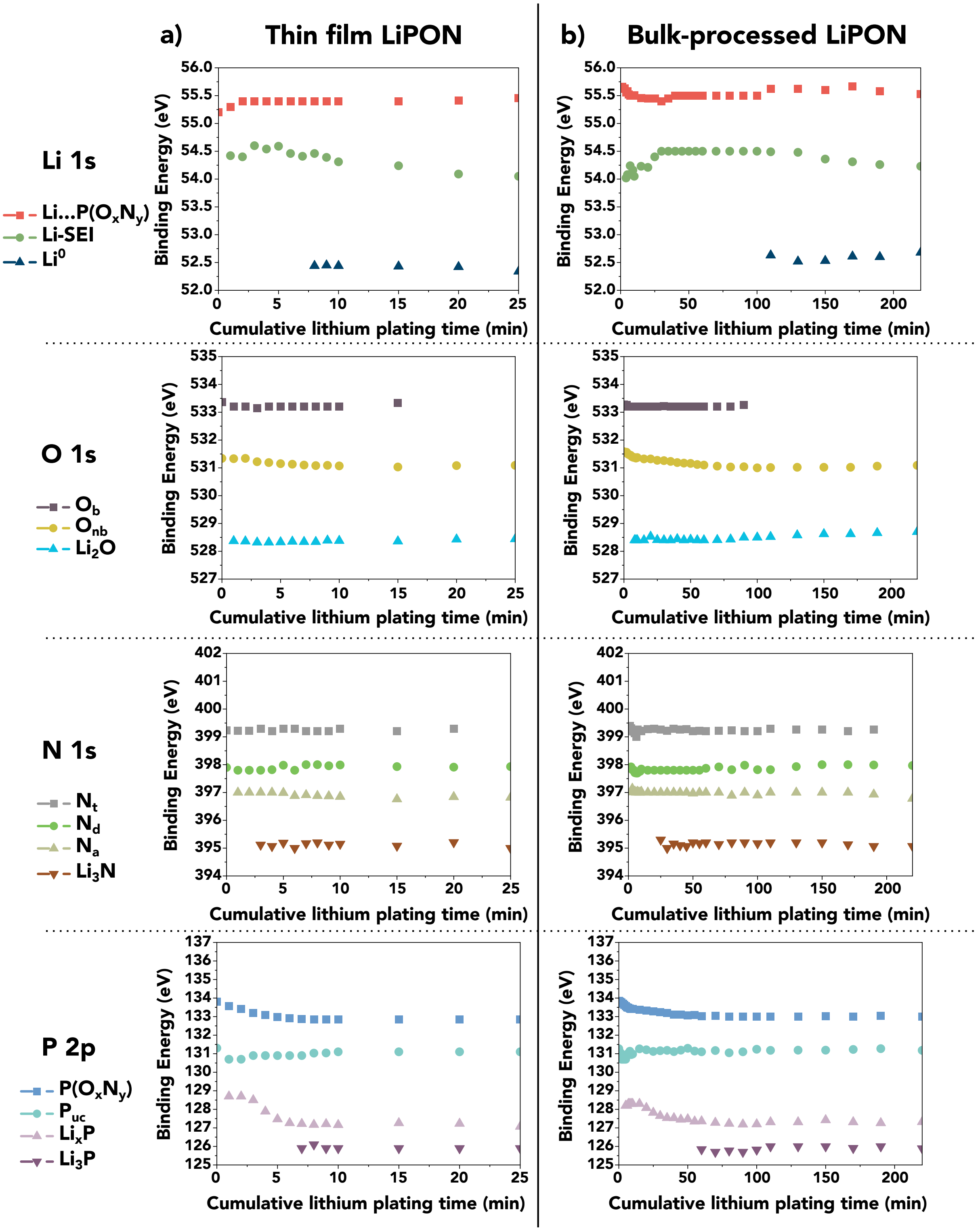

Figure S11: Evolution of the core-level XPS spectral component binding energies for a) the ∼0.6 µm thick LiPON film sample and b) the ∼800 µm thick bulk-processed LiPON sample during the *in situ* lithium plating experiments.

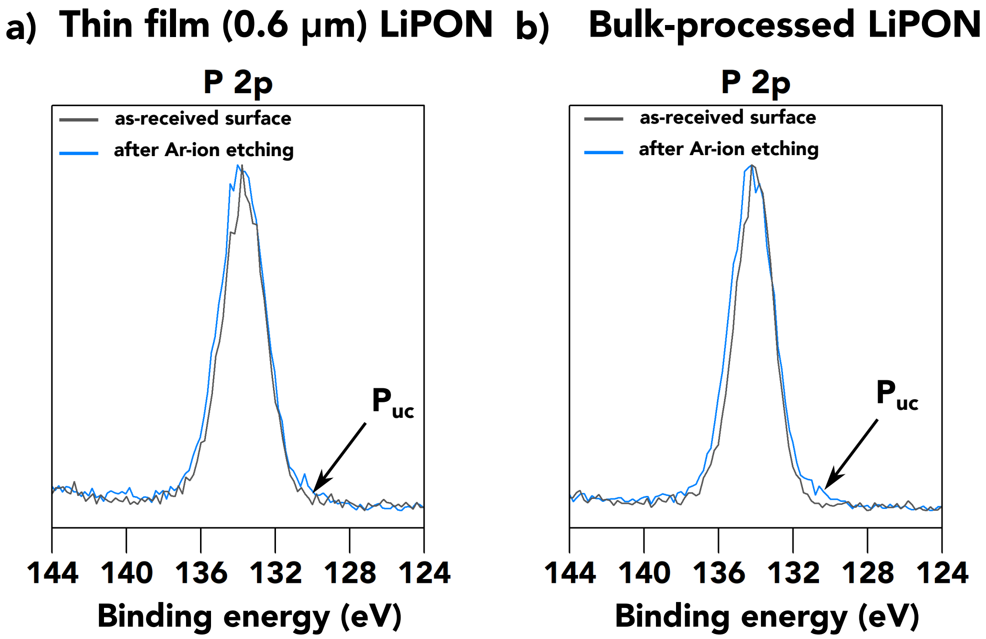

Figure S12: Normalized P 2p XPS spectra in the binding energy range of 144 eV to 124 eV extracted from survey scans for a) the ∼0.6 μm thick LiPON film sample and b) the ∼800 μm thick bulk-processed LiPON sample before and after Ar-ion etching. The arrows labelled "P_{uc}" point to possible emissions from undercoordinated phosphorus species.

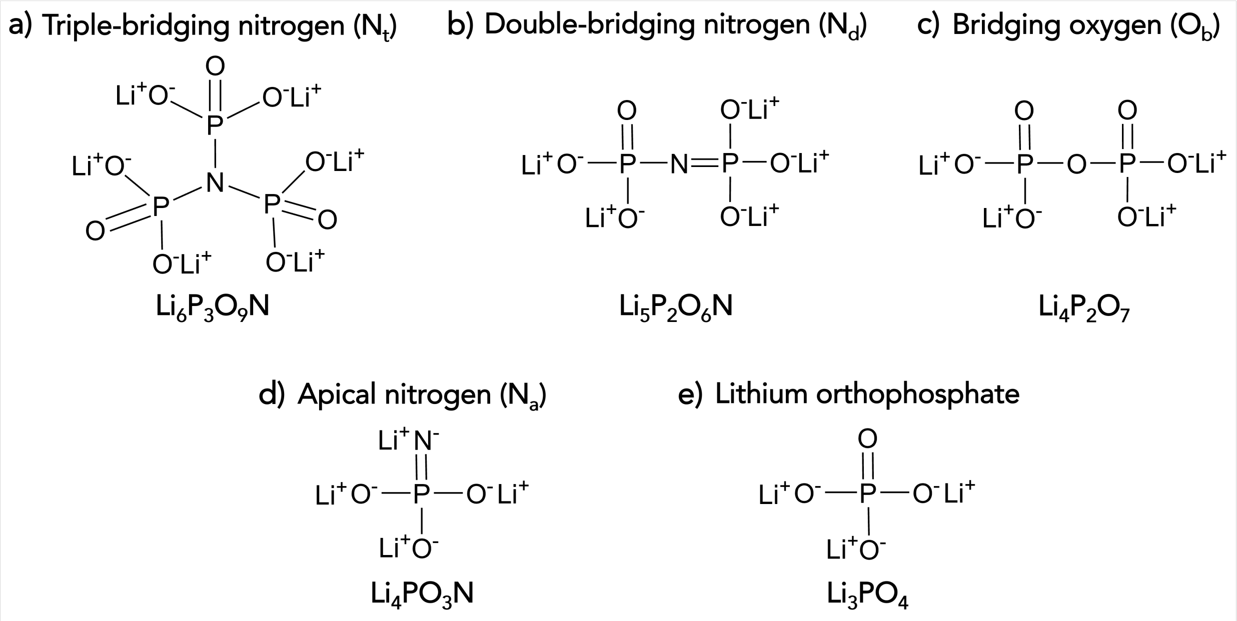

Figure S13: LiPON structural units (plus Li_3PO_4).

Table S3: Calculation of the minimum atomic fraction of P associated with Li_3PO_4 for the ~0.6 µm LiPON film sample at a cumulative plating time of 10 minutes.

X	Concentration in sample (at%)	P/X ratio in structural unit	Concentration of P (at%)
O_b	0.21	2	0.42
N_t	0.24	3	0.72
N_d	0.38	2	0.76
N_a	2.57	1	2.57
Sum [P]			4.47
$P(O_xN_y)$	5.66	1	5.66
Difference			1.19

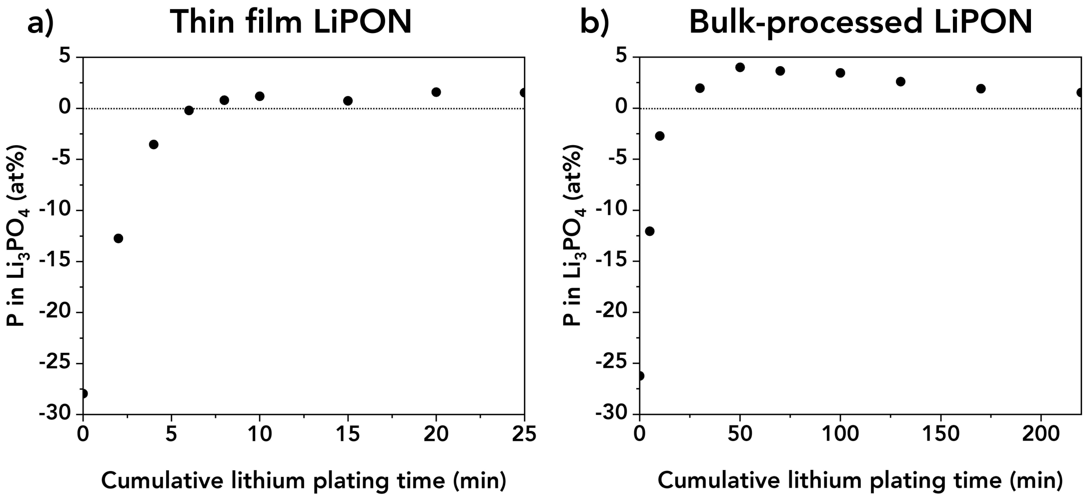

Figure S14: Evolution of the minimum (mathematically) possible concentration of P associated with Li_3PO_4 in a) the ~0.6 µm thick LiPON film sample and b) the ~800 µm thick bulk-processed LiPON sample with cumulative lithium plating time. Negative values are non-physical.

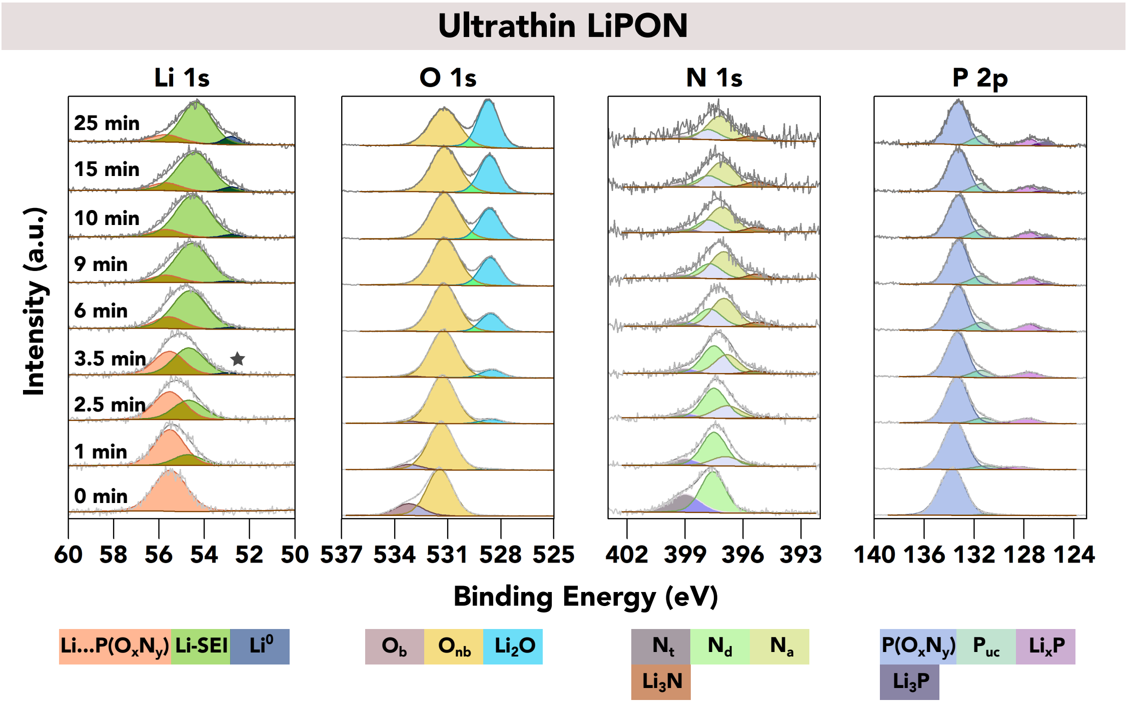

Figure S15: Time series of XPS core-level spectra acquired during the *in situ* lithium plating experiments performed on a ∼0.01 µm thick LiPON film sample. Acquired spectra (grey) are shown along with linear combination fitting results; the intensities were normalized to the strongest peak in each spectrum. The cumulative lithium plating time (electron beam exposure time) is indicated in the first column. For clarity, spectra acquired at intermediate plating times are not displayed. Individual spectral components are coloured, and a key is provided below each group of spectra. O_b, O_{nb}, N_t, N_d, N_a and P_{uc} are bridging oxygen, non-bridging oxygen, triple-bridging nitrogen, double-bridging nitrogen, apical (non-bridging) nitrogen and undercoordinated phosphorus, respectively. The appearance of the Li^0 (lithium metal) peak is highlighted with a star.

References

(1) Kaneko, S.; Yokomizo, M.; Mochizuki, Y.; Arashida, T.; Sasamoto, T.; Tanabe, T.; Matsumoto, F. Influence of N_2 Concentration in the Induction Gases on the Ionic Conductivity of Lithium Phosphorus Oxynitride Solid Electrolyte Thin Film Prepared by Magnetron Sputtering. *Journal of the Surface Finishing Society of Japan* **2015**, *66*, 540–543.
